# *APOE4*, Blood Neurodegenerative Biomarkers, and Cognitive Decline in Community-Dwelling Older Adults

**DOI:** 10.1001/jamanetworkopen.2025.8903

**Published:** 2025-05-07

**Authors:** Ted K. S. Ng, Todd Beck, Patricia Boyle, Klodian Dhana, Pankaja Desai, Denis A. Evans, Kumar B. Rajan

**Affiliations:** 1 Rush Institute for Healthy Aging, Rush University Medical Center, Chicago, Illinois; 2 Rush Alzheimer’s Disease Center, Rush University Medical Center, Chicago, Illinois

## Abstract

**Importance:**

Scarce population-based data exist on whether *APOE4* modifies associations of blood-based neurodegenerative biomarkers with cognitive decline, particularly in a diverse, biracial population of community-dwelling older adults without dementia.

**Objective:**

To assess whether *APOE4* carrier status is associated with an accelerated rate of cognitive decline in older adults without dementia and with elevated neurodegenerative burden.

**Design, Setting, and Participants:**

This 20-year prospective cohort study started in 1993 and was conducted through 2012 on the South Side of Chicago among community-dwelling older adults without dementia from the longitudinal biracial Chicago Health and Aging Project. The interaction of *APOE4* carrier status with prospective associations of serum neurodegenerative biomarkers with global cognitive decline was examined using a mixed-effects regression model, adjusting for demographics and chronic health conditions. Statistical analyses were conducted from June 2024 to January 2025.

**Exposure:**

*APOE4* carrier status and serum biomarker levels for total tau (t-tau), neurofilament light (NfL) chain, and glial fibrillary acidic protein (GFAP) measured with a Quanterix Neuroplex kit at baseline.

**Main Outcomes and Measures:**

Cognitive decline calculated from composite global cognition scores across study waves.

**Results:**

Among 1038 community-dwelling older adults (mean [SD] age, 77.1 [5.9] years; 615 Black [59.2%] and 423 White [40.8%]; 651 female [62.7%]), there was a mean (SD) of 12.8 (3.4) years of education and 343 individuals (33.0%) were *APOE4* carriers. Higher levels of blood-based neurodegenerative biomarkers (ie, t-tau, NfL, and GFAP) were associated with a faster rate of cognitive decline among *APOE4* carriers than noncarriers. Specifically, compared with noncarriers, *APOE4* carriers had annual rates of cognitive decline per 1–log_10_ unit higher levels in t-tau and GFAP that were accelerated by a β (SD) of −0.03 (0.02) (*P* = .046) and −0.07 (0.03) (*P* = .02), respectively. Similarly, compared with noncarriers and participants in the lower NfL tertile, *APOE4* carriers with middle and upper tertiles of NfL levels experienced accelerated cognitive decline, with a β (SD) of −0.04 (0.02) (*P* = .006) and −0.03 (0.02) (*P* = .07), respectively, although the difference was not significant for upper tertiles.

**Conclusions and Relevance:**

This study found that higher levels of neurodegeneration (t-tau), axonal injury (NfL), and reactive astrocytes and neuroinflammation (GFAP) biomarkers were associated with accelerated cognitive decline in genetically susceptible *APOE4* carriers. These findings highlight the association of *APOE4* with exacerbation of neurodegenerative processes, with not only significant implications for understanding and tracking the progression of neurodegenerative diseases, but also a call for inclusivity of *APOE4* status in scientific investigations and clinical trials.

## Key Points

**Question:**

Is the rate of cognitive decline in older adults without dementia and with elevated neurodegenerative burden accelerated in *APOE4* carriers?

**Findings:**

In this cohort study of 1038 community-dwelling older adults, higher levels of neurodegeneration (total tau), axonal injuries (neurofilament light), and reactive astrocytes and neuroinflammation (glial fibrillary acidic proteins) were associated with accelerated cognitive decline in genetically susceptible *APOE4* carriers without dementia compared with noncarriers.

**Meaning:**

These findings suggest the association of *APOE4* with exacerbated neurodegenerative processes, with not only significant implications for understanding and tracking the progression of neurodegenerative diseases, but also a call for inclusivity of *APOE4* status in scientific investigations and clinical trials.

## Teaser

This cohort study investigates the interaction of *APOE4* carrier status with the association between neurodegenerative biomarkers and rate of cognitive decline in older adults.

## Introduction

Alzheimer disease and related dementias (ADRDs) are multifactorial neurodegenerative disorders defined biologically by the ATN-I framework, characterized by the accumulation of β-amyloid plaques (A), tau tangles (T), neurodegeneration (N), and neuroinflammation (I).^1^ The *APOE4* allele is the most dominant genetic risk factor for late-onset AD^2,3,4,5^ and increases AD risk through several potential mechanisms, resulting in high amyloid burden, accumulation of tau tangles, and microglial dysfunction.^6,7^ Specifically, in the central nervous system, the apolipoprotein E protein is expressed by the glial cells, predominantly in the astrocytes.^8^

Apart from β-amyloid plaque (A) and tau tangle (T) AD biomarkers,^1^ AD blood-based neurodegenerative biomarkers include the total tau (t-tau) and glial fibrillary acidic proteins (GFAPs), which are indicative of neurodegeneration (N) and neuroinflammation (I) in the central nervous system, respectively, and neurofilament light (NfL), which reflects non–AD-specific subcortical, large-caliber axonal degeneration.^8,9,10,11^ These blood-based neurodegenerative markers have been shown to predict cognitive decline robustly and longitudinally years before cognitive decline in human participants.^12,13,14,15,16,17^

However, although recent studies have acknowledged the importance of *APOE4* by including it as a covariate,^18,19,20,21^ scarce population-based data exist on whether *APOE4* modifies the associations of blood-based neurodegenerative biomarkers with cognitive decline, particularly in diverse, biracial populations of community-dwelling older adults without dementia. For example, only 2 studies, both published in the past year, have examined *APOE4*’s modification of the associations of 2 select blood-based AD and neurodegenerative markers, plasma p-tau and NfL levels.^22,23^ One of the studies was a small clinical study^23^ on the associations of plasma p-tau181, p-tau231, and GFAP with cognitive decline. The study investigated cross-sectional but not prospective associations of the biomarkers with cognitive decline.^23^ In studies on animals with the astrocyte-specific GFAP promoter compared with APOE3-secreting astrocytes, there was a significantly decreased number of neurite outgrowths from neurons cultured with *APOE4*-secreting or APOE-knockout astrocytes.^24^ Despite these findings, to our knowledge, no population-based study has investigated the modification of GFAP associations by *APOE4* carrier status. Furthermore, to the best of our knowledge, no studies on *APOE4*’s modification of the associations of t-tau levels with cognitive decline, specifically in community-dwelling older adults, have been performed.

Additionally, more diverse demographics are needed to enhance the generalizability of blood-based biomarker studies. Extant studies are among overwhelmingly White populations, excluding members of racial minority groups, who have been estimated to have a higher risk for and the steepest increase in incident ADRD in the next 40 years,^25,26^ as well as a higher frequency of the *APOE4* allele due to genetic inheritance and ancestry.^27^ Furthermore, many blood-based biomarker studies recruited human participants solely from clinical settings, often with very different demographics and AD risk than real-world, community-dwelling older adults.

To address these gaps, we hypothesized that the combination of *APOE4* carrier status and increased blood-based neurodegenerative marker levels, indicated by higher levels of blood-based neurodegeneration (t-tau), axonal injury (NfL), and reactive astrocytes and neuroinflammation (GFAP), would constitute a higher neurodegenerative burden and neuronal damage in *APOE4* carriers than noncarriers. We hypothesized that this would be associated with an accelerated rate of cognitive decline in a biracial population of community-dwelling older adults without dementia.

## Methods

### Study Participants

The Chicago Health and Aging Project (CHAP) enrolled participants based on a door-to-door census in 4 Chicago neighborhoods with substantial proportions of Black older adults and White residents.^28,29^ Inclusion criteria required that participants live in the geographical areas and be aged older than 65 years. CHAP data were collected in triennial cycles, with the baseline cohort starting in 1993 and subsequent cycles in 1997, 2000, 2003, 2006, 2009, and 2012. In each cycle, a stratified random sample based on age, sex, race, and cognitive level was selected for clinical evaluation of incident AD dementia when the participants also provided blood samples. Starting in the fifth cycle, all older adults who consented to blood sample collection also provided blood samples. Participants who had not consented to blood sample collection in the fifth cycle but had consented in subsequent cycles were included. For this study, we restricted the population to individuals fulfilling the following additional criteria: (1) participants had no dementia at study baseline, indicated by the absence of a clinical diagnosis of AD performed as part of the clinical assessment or a cutoff of Mini-Mental State Examination (MMSE) score of 24 or greater for individuals without a clinical diagnosis (ie, for participants who were not randomly selected to participate in the clinical assessment component); (2) participants provided blood samples in 1 cycle; and (3) participants had 1 or more cycles of composite global composite cognitive scores. Across study waves, 1038 participants without dementia fulfilled all criteria and were included in this study (eFigure 1 in Supplement 1). This study is reported following the Strengthening the Reporting of Observational Studies in Epidemiology (STROBE) reporting guideline for cohort studies. The CHAP study was approved by the Institutional Review Board of Rush University Medical Center, and each participant provided written informed consent.

### Quantification of Biomarker Serum Levels

We have described the blood specimen collection, selection, and processing in detail.^30,31^ In mid-2019, previously unthawed blood sample aliquots were shipped in dry ice to Quanterix Corporation. We focused on 3 neurodegenerative biomarkers: t-tau, NfL, and GFAP. They were assayed using single-molecule ultrasensitive immunoassays in duplicates with several control assays in a Quanterix bead-based HD platform and Neurology 4-Plex kit. The coefficient of variation in duplicate samples was 7.3% for t-tau and 3.0% for NfL and GFAP. For our analysis, we found the mean of duplicate measurements for each participant and used the mean level of biomarkers.

### APOE Genotypes

APOE genotypes were ascertained using 2 single-nucleotide variants (SNVs; formerly single-nucleotide polymorphisms): rs7412 and rs429358.^32,33^ We genotyped the 2 SNVs at the Broad Institute Center for Genotyping using the homogeneous mass extension Sequenom MassArray platform. Genotyping call rates were 100% for SNV rs7412 and 99.8% for SNV rs429358. Both SNVs were in Hardy-Weinberg equilibrium, with *P* values of .08 and .79, respectively. Based on these 2 SNVs, we created an indicator variable for participant *APOE4* carrier status.^34,35^ Participants with ε2/ε4, ε3/ε4, and ε4/ε4 were classified as *APOE4* carriers, and those with ε2/ε2 ε2/ε3, and ε3/ε3 genotypes were classified as non–*APOE4* carriers.

### Global and Individual Tests of Cognition

We administered 4 brief performance tests of cognitive function at 3-year intervals as part of a structured in-home population interview, including immediate and delayed recall tests, the Symbol Digit Modalities Test,^36^ and the MMSE.^37^ Immediate and delayed recall of 12 ideas in the East Boston Story^38^ provided measures of episodic memory. The oral version of the Symbol Digit Modalities Test^28^ was administered to assess perceptual speed, a component of executive function. The MMSE^37^ provided a measure of global cognition. Because in a previous factor analysis, all 4 cognitive tests loaded on a single factor that accounted for 75% of the variance,^39^ we formed a composite measure of global cognition based on the 4 tests. As previously described, raw scores on each test were converted to *z* scores using baseline means and SDs of the entire CHAP population, and we found means of *z* scores to yield the composite global cognition score.^39^ We used the composite as the primary outcome because composite measures minimize floor and ceiling artifacts. Individual tests of cognition were also conducted in sensitivity analyses.

### Demographic Variables and Other Covariates

During the baseline assessment, we collected demographic measures: age at the time of first blood sample collection, biological sex (male or female), and self-reported race (Black older adults and White older adults) using items from the 1990 US Census Bureau^40^ and education (measured in the number of years of schooling completed). Our analysis centered on baseline age at 75 years and education at 12 years and included indicator variables for female sex (reference group, male) and Black older adults (reference group, White older adults). Other covariates included common chronic conditions (ie, heart disease, diabetes, hypertension, and stroke), defined by self-report questions from the Established Populations for the Epidemiologic Study of the Elderly,^41^ as well as body mass index (BMI; calculated as weight in kilograms divided by height in meters squared).

### Statistical Analysis

Baseline descriptive statistics were computed for demographic and cognitive characteristics, including age, number of formal years of education completed, self-reported race, sex at birth, and global cognition, and stratified by the *APOE4* allele. Levels of blood biomarkers were positively skewed and greater than zero. Hence, we made a log_10_ transformation with geometric means and their 95% CIs. Descriptive comparisons between participants with and without the *APOE4* were based on *t* tests for untransformed characteristics, χ^2^ tests for frequencies, and the Wilcoxson rank test for serum biomarkers. All regression models were adjusted for age at first blood sample collection (centered at 75 years), education (centered at 12 years), female sex, Black older adult race, BMI, and common chronic conditions, including heart disease, diabetes, hypertension, stroke.

We used linear mixed-effects regression modeling to examine the association of baseline blood-based biomarker levels with the baseline level of cognitive function and longitudinal changes in the annual rate of cognitive decline, with intercept and slope. Model assumptions were checked analytically and graphically and were deemed to be adequately met, including linearity, homoscedasticity, normality of residuals, and multicollinearity.

Time since baseline blood assessment in years captured the annual rate of change in cognitive function over time. We operationalized biomarkers in 2 different ways. First, we used log-transformed continuous biomarker levels. Second, we categorized tertiles of serum biomarker levels with 2 indicator variables for the second and third tertiles (with the first tertile as the reference group) to examine the association of higher tertiles of biomarker levels with cognitive decline.

We also performed 2 separate yet related sets of analyses. First, we ran a 3-way interaction model incorporating the interaction term *APOE4 carrier status × time × continuous biomarker levels/tertiles*. Subsequently, we stratified the total sample by *APOE4* carrier status. Between– and within–*APOE4* carrier differences were tested using contrast for estimates from respective regression models.

All regression models were performed using the nlme library, and graphical representations were performed with R statistical software version 4.2 (R Project for Statistical Computing).^42^ *P* values < .05 were considered statistically significant, and *P* values were 2-sided. We did not perform adjustments for multiple comparisons given that all analyses were based on an a priori hypothesis and contrasts were based on a single regression model for each biomarker. Statistical analyses were conducted from June 2024 to January 2025.

Similarly, using linear mixed-effects regression modeling, we performed 3 sets of sensitivity analyses to test the robustness of conclusions and granularity in the data that may suggest subgroup differences:

1. After removing baseline cases of mild cognitive impairment, the total sample was further reduced to include 802 participants without any cognitive impairment.
2. Instead of a composite cognitive measure, individual domains or tests of cognition (ie, episodic memory, perceptual speed and executive function, and MMSE) were evaluated.
3. Finally, individual vs combined neurodegenerative markers and their differential associations with cognitive decline were assessed.

## Results

### Sample Characteristics

Among 1038 participants (mean [SD] age, 77.1 [5.9] years; 615 Black [59.2%] and 423 White [40.8%]; 651 female [62.7%]), there was a mean (SD) of 12.8 (3.4) years of education, 343 individuals (33.0%) were *APOE4* carriers, and 695 individuals were non–*APOE4* carriers (67.0%) (Table 1). There was a mean (SD) of 6.5 (3.9) years of follow-up. The distribution of *APOE4* genotypes in the total sample, showing a low number of *APOE4* homozygotes, is shown in eTable 1 in Supplement 1. At baseline, *APOE4* carriers compared with non–*APOE4* carriers were a year younger (mean [SD] age, 76.4 [5.4] years vs 77.4 [6.1] years; *P* = .02) and had a greater proportion of Black older adults (226 Black participants [66.X%] vs 389 Black participants (56.X%]; *P* = .003) but had comparable education levels and global cognition scores. *APOE4* carriers also had higher mean (SD) baseline levels of 2 neurodegenerative biomarkers compared with noncarriers: 0.82 (2.48) pg/mL vs 0.67 (3.03) pg/mL for t-tau, for a difference of 0.15 pg/mL (*P* = .002), and 277.8 (195.6) pg/mL vs 251.4 (178.0) pg/mL for GFAP, for a difference of 26 pg/mL (*P* = .03). Conversely, NfL levels were not significantly different between *APOE4* carriers and noncarriers (Table 1; eFigure 2 in Supplement 1).

**Table 1.** Baseline Characteristics

Characteristic	Participants, No. (%)			*P* value^a^
	Overall (N = 1038)	Non–*APOE4* carriers (n = 695)	*APOE4* carriers (n = 343)	
Age, mean (SD), y	77.1 (5.9)	77.4 (6.1)	76.4 (5.4)	.02
Education, mean (SD), y	12.8 (3.4)	12.8 (3.5)	12.8 (3.3)	.84
Composite global cognition, mean (SD)^b^	0.34 (0.55)	0.36 (0.55)	0.30 (0.55)	.07
Race				
Black	615 (59.2)	389 (55.9)	226 (65.9)	.003
White	423 (40.8)	306 (44.1)	117 (34.1)	
Sex				
Female	651 (62.7)	443 (63.7)	208 (60.6)	.37
Male	387 (37.3)	252 (35.3)	135 (39.4)	
Chronic health conditions				
Heart disease	138 (13.3)	99 (14.2)	39 (11.4)	.24
Diabetes	230 (22.2)	156 (22.4)	74 (21.6)	.81
Hypertension	625 (60.2)	402 (57.8)	223 (65.0)	.03
Stroke	93 (9.0)	63 (9.1)	30 (8.7)	.96
BMI, mean (SD)	27.3 (5.3)	27.4 (5.4)	27.2 (5.1)	.71
Serum biomarker levels, mean (SD), pg/mL				
T-tau	0.72 (2.86)	0.67 (3.03)	0.82 (2.48)	.002
NfL	33.5 (51.1)	31.8 (42.9)	36.9 (64.5)	.48
GFAP	260.1 (184.3)	251.4 (178.0)	277.8 (195.6)	.03

Abbreviations: BMI, body mass index (calculated as weight in kilograms divided by height in meters squared); GFAP, glial fibrillary acidic protein; NfL, neurofilament light chain; t-tau, total tau.

^a^ Comparisons were based on the Welch 2-sample *t* test for untransformed characteristics, Pearson χ^2^ test for frequencies, and Wilcoxon rank sum test for serum biomarker levels.

^b^ A composite measure of global cognition based on 4 tests: immediate and delayed recall tests, the Symbol Digit Modalities Test, and the Mini-Mental State Examination.

### Interactions Between *APOE4* Carrier Status and Associations of Biomarkers With Cognitive Decline

#### Linear Association

There were 3-way interactions in t-tau and GFAP models (Table 2).For APOE 4 × time × t-tau, the estimate (SD) was −0.03 (0.02) (*P* = .046), and for APOE 4 × time × GFAP, the estimate (SD) was −0.07 (0.03) (*P* = .02). Conversely, there were no significant 3-way interactions in the NfL model.

**Table 2.** Interaction of *APOE4* Carrier Status With Associations Between Biomarkers and Cognitive Decline^a^

Variable	T-tau		NfL		GFAP	
	Estimate (SD)	*P* value	Estimate (SD)	*P* value	Estimate (SD)	*P* value
**Biomarker linear, pg/ml**						
Biomarker^b^	0.02 (0.04)	.57	−0.12 (0.07)	.11	−0.13 (0.08)	.13
Intercept	0.54 (0.04)	<.001	0.69 (0.11)	<.001	0.82 (0.20)	<.001
*APOE4* carrier status	−0.03 (0.04)	.49	0.19 (0.16)	.25	0.01 (0.31)	.98
Time	−0.04 (0.01)	<.001	−0.002 (0.02)	.94	0.03 (0.04)	.53
*APOE4* × time × biomarker^b^	−0.03 (0.02)	.046	−0.03 (0.03)	.28	−0.07 (0.03)	.02
**Biomarker tertiles**						
Lower tertile^c^	Reference	NA	Reference	NA	Reference	NA
Middle tertile^d^	−0.002 (0.04)	.90	−0.02 (0.04)	.70	−0.02 (0.04)	.58
Upper tertile^e^	−0.02 (0.04)	.59	−0.09 (0.05)	.07	−0.08 (0.05)	.10
Intercept	0.54 (0.04)	<.001	0.55 (0.04)	<.001	0.56 (0.04)	<.001
*APOE4* carrier status	−0.08 (0.05)	.12	0.02 (0.05)	.67	−0.03 (0.05)	.53
Time	−0.04 (0.01)	<.001	−0.04 (0.01**)**	<.001	−0.03 (0.01)	<.001
*APOE4* × time × lower tertile biomarker	[Reference]	NA	[Reference]	NA	[Reference]	NA
*APOE4* × time × middle tertile biomarker	−0.002 (0.02)	.89	−0.04 (0.02)	<.01	−0.004 (0.02)	.79
*APOE4* time × upper tertile biomarker	−0.04 (0.02)	.045	−0.03 (0.02)	.07	−0.03 (0.02)	.07

Abbreviations: GFAP, glial fibrillary acidic protein; NA, not applicable; NfL, neurofilament light chain; t-tau, total tau.

^a^ Linear mixed-effects regression models were conducted among 1033 participants (5 participants were not included in the model due to missing ≥1 covariates) and adjusted for age (centered at 75 years), education (centered at 12 years), female sex, Black older adult race, body mass index (calculated as weight in kilograms divided by height in meters squared), and chronic conditions and included the interaction of these characteristics with time since baseline.

^b^ Biomarker variables in rows match column biomarkers, with t-tau for t-tau estimates, NfL for NfL estimates, and GFAP for GFAP estimates.

^c^ Lower tertile was less than 0.26 pg/mL among 378 participants for t-tau, less than 20.5 pg/mL among 389 participants for NfL, and less than 180 pg/mL among 373 participants for GFAP.

^d^ Middle tertile was 0.26 to 0.56 pg/mL among 348 participants for t-tau, 20.5 to 31.5 pg/mL among 347 participants for NfL, and 180 to 300 pg/mL among 356 participants for GFAP.

^e^ Upper tertile was greater than 0.56 pg/mL among 309 participants for t-tau, greater than 31.5 pg/mL among 298 participants for NfL, and greater than 300 pg/mL among 305 participants for GFAP.

#### Associations of Biomarker Tertiles

Similar to the linear associations, when each biomarker was operationalized as tertiles, 3-way interaction terms remained significant for both tertiles of t-tau and GFAP (eTable 4 in Supplement 1). Contrary to the linear association, the 3-way interaction model with the middle tertile NfL was significant; for APOE 4 × time × middle tertile NfL, the estimate (SD) was −0.04 (0.02) (*P* = .006); for the upper tertile, the outcome was not significant (−0.03 [0.02]; *P* = .07).

### Associations of Biomarker Levels With Cognitive Decline by *APOE4* Carrier Status

#### Linear Association

Within groups, the rate of cognitive decline was not accelerated in non–*APOE4* carriers but was accelerated among *APOE4* carriers per unit increase in t-tau, with an estimate (SD) of −0.04 (0.02) per year (*P* = .02) (eTable 2 in Supplement 1). Similarly, the rate of cognitive decline was not accelerated in non–*APOE4* carriers, but it was accelerated among *APOE4* carriers per unit increase in NfL, with an estimate (SD) of −0.06 (0.03) per year (*P* = .02) (Table 3). For GFAP, as with the other 2 biomarkers, the rate of cognitive decline was not accelerated in non–*APOE4* carriers, but it was accelerated among *APOE4* carriers per unit increase in GFAP, with an estimate (SD) of −0.12 (0.03) per year (*P* < .001) (eTable 2 in Supplement 1).

**Table 3.** Associations of NfL Levels With Annual Rate of Cognitive Decline^a^

NfL measure	Non–*APOE4* carriers (n = 693)		*APOE4* carriers (n = 340)	
	Estimate (SD)	*P* value	Estimate (SD)	*P* value
Linear, pg/mL	−0.11 (0.08)	.17	−0.30 (0.10)	<.01
Intercept	0.67 (0.11)	<.001	0.90 (0.15)	<.001
Time	0.0004 (0.02)	.98	−0.02 (0.04)	.69
NfL × time	−0.02 (0.02)	.18	−0.06 (0.03)	.02
Tertile group^b^				
Lower tertile	Reference	NA	Reference	NA
Middle tertile	−0.01 (0.04)	.77	−0.05 (0.06)	.36
Upper tertile	−0.07 (0.05)	.13	−0.27 (0.07)	<.001
Intercept	0.55 (0.04)	<.001	0.58 (0.07)	<.001
Time	−0.03 (0.008)	<.001	−0.07 (0.02)	<.001
Lower tertile × time	[Reference]	NA	[Reference]	NA
Middle tertile × time	0.01 (0.01)	.29	−0.05 (0.02)	<.01
Upper tertile × time	−0.01 (0.01)	.28	−0.06 (0.02)	<.01

Abbreviations: NA, not applicable; NfL, neurofilament light chain.

^a^ The linear mixed-effects regression models were conducted among 1033 participants and adjusted for age (centered at 75 years), education (centered at 12 years), female sex, Black older adult race, body mass index (calculated as weight in kilograms divided by height in meters squared), and chronic conditions and included the interaction of these characteristics with time since baseline.

^b^ Lower tertile was less than 20.5 pg/mL among 269 noncarriers and 120 carriers. Middle tertile was 20.5 to 31.5 pg/mL among 222 noncarriers and 125 carriers. Upper tertile was greater than 31.5 pg/mL among 201 noncarriers and 97 carriers.

#### Associations of Biomarker Tertiles

**Figure 1.**
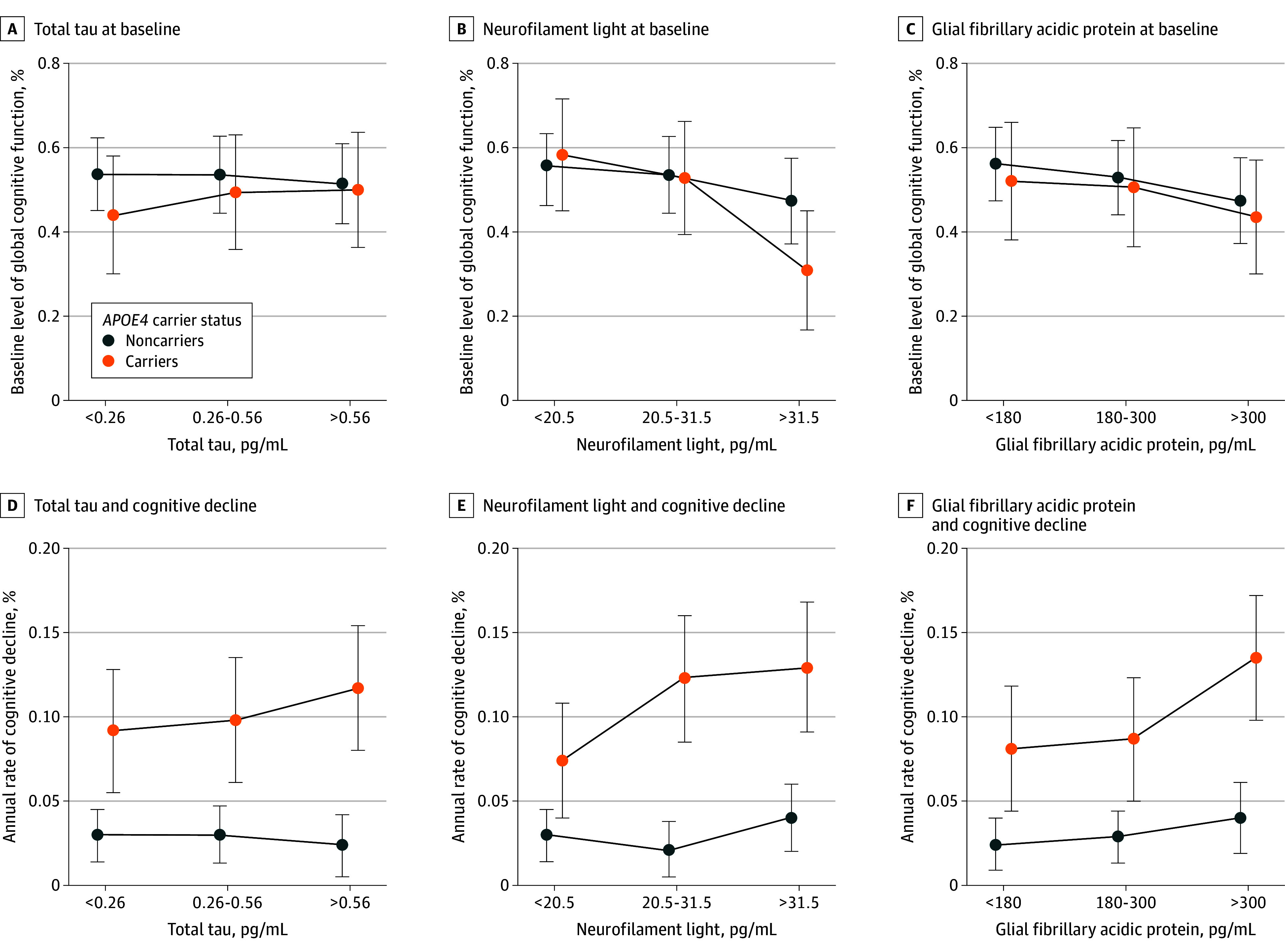
Estimated Baseline Levels of Cognitive Function and Annual Rate of Cognitive Decline Estimated measures are given with 95% CIs by serum neurodegenerative biomarker tertile among *APOE4* carriers vs noncarriers. Compared with noncarriers, *APOE4* carriers had lower baseline levels of cognitive function and accelerated annual rates of cognitive decline, especially those with upper tertiles of biomarkers. Estimates are derived from the interaction of biomarkers with time since baseline in linear mixed-effects regression models adjusting for demographic and chronic health conditions. Whiskers indicate 95% CIs.

**Figure 2.**
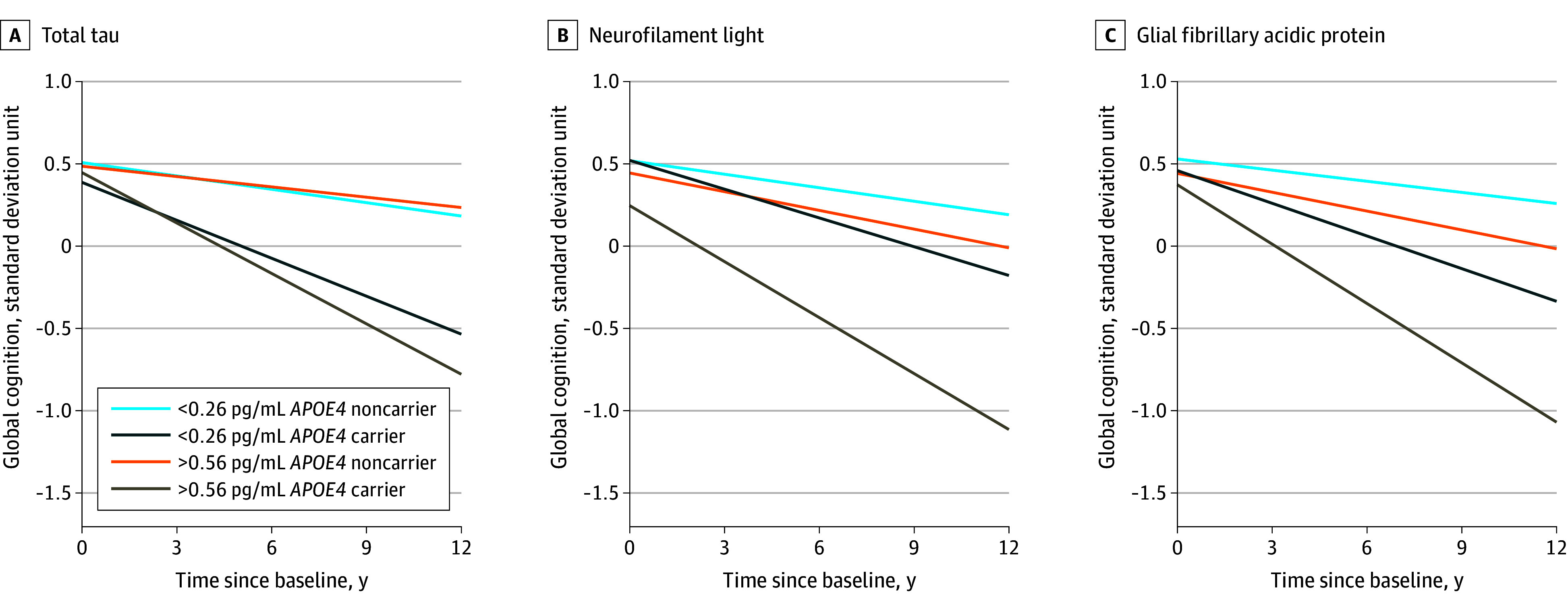
Longitudinal Cognitive Decline in Lower and Upper Tertiles of Biomarkers Models were adjusted for demographic and chronic health conditions to examine the interaction between *APOE4* carrier status and the combined associations of biomarkers with global cognitive function.

Within groups, the rate of cognitive decline was not accelerated in non–*APOE4* carriers or *APOE4* carriers with increased t-tau levels (eTable 2 and eFigure 3 in Supplement 1; Figure 1 and Figure 2). Conversely, the rate of cognitive decline was not accelerated in non–*APOE4* carriers but was accelerated among *APOE4* carriers with NfL in middle and upper tertiles, with an estimate (SD) of −0.05 (0.02) (*P* = .003) and −0.06 (0.02) (*P* = .004), respectively (Table 3; Figure 1 and Figure 2; eFigure 3 in Supplement 1). Similarly, the rate of cognitive decline was accelerated in only *APOE4* carriers with upper tertile GFAP levels (eTable 2 and eFigure 3 in Supplement 1; Figure 1 and Figure 2). Models also examined cross-sectional associations between the 3 biomarkers and baseline cognitive score and found similar associations (Figure 1).

### Sensitivity Analyses

Results for all sensitivity analyses are presented in eTables 3, 4, and 5 in Supplement 1. First, we added a sensitivity analysis on the total sample excluding individuals with mild cognitive impairment or cognitive impairment (eTable 3 in Supplement 1). Results and conclusions remain unchanged. Second, individual domains or tests of cognition were also assessed in addition to a composite cognitive measure (eTable 4 in Supplement 1). We found that associations were driven by memory domains given that there was a lack of associations with executive function regardless of *APOE4* status.

Lastly, we performed another set of sensitivity analyses examining associations of multiple high levels of these neurodegenerative biomarkers with cognitive decline (eTable 5 in Supplement 1). Compared with participants with only a single elevated biomarker (the reference group), those with *combined biomarker middle tertile* (ie, all 3 biomarkers in the middle tertiles) who were also *APOE4* carriers experienced an even faster rate of cognitive decline. Participants with *combined biomarker upper tertile* (ie, all 3 biomarkers in the upper tertiles) who were also *APOE4* carriers experienced the fastest (ie, worst) rate of cognitive decline.

## Discussion

In this cohort study, compared with non–*APOE4* carriers, neurodegenerative serum biomarkers of t-tau, NfL, and GFAP were associated with lower baseline cognitive function levels and a faster cognitive decline rate among *APOE4* carriers without dementia. Notably, when considering both baseline neurodegenerative serum biomarkers and the *APOE4* carrier status, the difference in cognitive decline between *APOE4* carriers and noncarriers increased with higher levels of t-tau, NfL, or GFAP. Taken together, our findings suggest that the combined elevated neurodegenerative burdens imposed by *APOE4* and neurodegenerative biomarkers were associated with an accelerated rate of cognitive decline in a biracial population of community-dwelling older adults without dementia compared with associations of either *APOE4* or neurodegenerative biomarker alone. Our finding is of great public health significance given that we showed that an individual with the same level of neurodegenerative serum biomarkers could have a very different risk based on their *APOE4* carrier status. Hence, risk predictions based on these blood-based neurodegenerative biomarkers alone are likely insufficient. This prompts the need to concurrently account for *APOE4* carrier status to more accurately monitor differences in risk of cognitive decline and, by extension, the risk of ADRD in future scientific investigations and clinical trials.

These findings suggest that the influence of the *APOE4* allele may occur through several mechanisms.^43^ Given that blood t-tau level is a marker for neurodegeneration,^44^ a faster cognitive decline in *APOE4* carriers with high levels of t-tau suggests the association of *APOE4* with neurodegeneration, potentially amplifying tau’s pathological association with cognitive decline. The difference in cognitive decline in individuals with high t-tau levels was almost doubled in *APOE4* carriers compared with noncarriers. These findings indicate that the role of *APOE4* was more pronounced among individuals at a greater risk for neurodegeneration than those with a lower risk.

NfL is a nonspecific AD biomarker often associated with vascular aspects of dementia and indicative of the neurodegenerative processes of axonal injuries.^9,45,46,47,48^ Our findings demonstrated that *APOE4* carriers with higher NfL levels had a significantly greater rate of cognitive decline than noncarriers with the same NfL levels. Hence, older adults free of dementia but with higher neuronal injury had a much greater risk of accelerated cognitive decline and of developing dementia in the presence of the *APOE4* allele. As the biomarker levels increased, the rate of cognitive decline was substantially higher among *APOE4* carriers than noncarriers. These findings indicate that the influence of the *APOE4* allele may also occur through the cerebrovascular pathway of the disease, as observed through the NfL biomarker, which may be downstream of the neuronal injury pathway. Notably, a 2024 study^22^ found that almost all *APOE4* homozygotes exhibited AD pathology and had significantly higher levels of AD biomarkers, including cerebrospinal fluid Aβ_1-42_ and p-tau, amyloid-β as measured by positron emission tomography, plasma p-tau, and plasma NfL, suggesting that the *APOE4* may serve as a proxy for AD pathology. Hence, taken together with previous findings, our findings suggest that AD-associated pathology, conferred by *APOE4*, may interact with non–AD-specific pathology, conferred by NfL, in the association with accelerated cognitive decline.

Similarly, we found a significantly faster rate of cognitive decline in *APOE4* carriers with higher blood-based GFAP levels. The *APOE4* is primarily expressed by the astrocytes and upregulated by reactive microglia in the diseased state. The influence of the *APOE4* allele on astrocytes and of microglial dysfunction markers on cognitive decline, especially among humans, could thus be clinically significant. Although mechanistic evidence has been shown in animal models,^24,49^ our findings are, to the best of our knowledge, the first to report such an effect modification of the *APOE4* allele on neuroinflammation mediated by astrocytes and microglial dysfunction in the central nervous system in humans, specifically in a study of a large and diverse, biracial, community-based population of humans comprising more than 59% Black older adults and 63% females.

To test the robustness of and enhance the granularity in findings, we performed several sensitivity analyses. Taken together with the main analyses, these sensitivity analyses provided additional granularity to subgroup differences in the associations that were driven by cognitive domains and combinations of neurodegenerative burdens imposed by multiple biomarkers. Our data covered only 2 cognitive domains. Future studies with data on other cognitive domains should further examine variations in the associations. Notably, *APOE4* carriers with all 3 biomarkers in the upper tertiles also experienced the fastest (ie, worst) rate of cognitive decline. This finding suggests that multiple neurodegenerative pathways may be additive and associated with an even greater increase in risk of cognitive decline than a single neurodegenerative pathway.

### Strengths and Limitations

Our study has several notable strengths. First, our cohort comprised population-based community-dwelling older adults without dementia, which is much more representative of the larger at-risk population than clinical samples, which are typically restricted in demographics and more enriched in AD pathology. Hence, our findings may be more generalizable to the population of cognitively healthy and at-risk community-dwelling older adults, which forms a much larger portion of the older adult population. Second, our sample is unique in its biracial composition, with an overrepresentation of Black older adults, who are at a higher risk of developing AD and ADRD than White older adultss.^25^ This racially diverse population composition starkly contrasts with most cohort samples, which consist of predominantly White older adults, thus further enhancing the generalizability of our findings. Third, our cohort had a mean follow-up of 6.5 years, with frequent follow-up waves, enabling us to track cognitive decline over an extended period. Long-term follow-up is essential for tracking cognitive decline in older adults with dementia, among whom this decline could take many more years to manifest than among those with existing cognitive impairment or ADRD. Fourth, to our knowledge, this is the first population-based prospective study to have examined interactions and associations among 3 primary constructs (*APOE4* carrier status, blood-based neurodegenerative biomarkers, and measures of cognitive decline), notably investigating 3 serum neurodegenerative biomarkers and the modifying interactions of *APOE4* status with them in the same study, thus translating and validating animal findings in human samples. Furthermore, we excluded participants with clinician-diagnosed AD or MMSE-defined cases of probable AD or related dementias at baseline and further supplemented main analyses with sensitivity analyses excluding participants with mild cognitive impairment. This reduced the heterogeneity in biomarker levels and cognitive status, minimizing confounding effects imposed by existing AD comorbidities that would have otherwise skewed findings. Additionally, we performed a series of sensitivity analyses, testing the robustness of and enhancing the granularity in findings.

A few limitations may have also impacted our interpretations. Due to the low numbers of *APOE4* homozygotes, we focused analyses on *APOE4* carriers vs noncarriers. Owing to sample availabilities, we are restricted to analyzing serum instead of plasma or cerebrospinal fluid samples, focusing on 3 neurodegenerative blood-based biomarkers, and were not able to include other minority populations, such as American Indian, Asian, or Hispanic populations. Comparing blood biomarkers with postmortem neuropathological markers may provide additional details on the potential pathways of the *APOE4* association with AD and related dementias. Notably, we assayed serum biomarkers before the advent and validations of various phospho(p)-tau assays; future studies examining these neurodegenerative and neuroinflammation biomarkers and the amyloid and latest p-tau species in plasma, which are more specific to AD pathways, could validate and provide further complementary evidence specific to the AD neurodegenerative process. Indeed, we are currently assessing amyloid β and p-tau biomarkers in our newly collected plasma samples.

## Conclusions

Findings in this cohort study highlighted that in *APOE4* carriers compared with noncarriers, higher levels of neurodegenerative blood biomarkers were associated with a faster rate of cognitive decline in a biracial population of community-dwelling older adults without dementia. These findings have potential public health and clinical significance. First, our and others’ findings have shown that Black older adults have a greater frequency of the *APOE4* allele than White older adults^50,51,52^ and thus a greater risk of developing ADRD than those without the *APOE4* allele. Hence, our samples composition of 59% Black older adults enables the generalizability of the findings. Second, biomarkers of neurodegeneration and neuroinflammation are used for staging and prognosis and as indicators of biological treatment effect.^22^ They also have broader applications beyond cognitive decline caused by AD and ADRD and can be broadly applicable to various neurodegenerative diseases. Hence, our findings provide complementary and broader neurodegenerative disease mechanisms in older adults without cognitive impairment of 2 major races in the US. Particularly, serum samples have been widely collected in health care systems. Hence, our findings, assessed using serum samples could enhance the broader adoption of blood-based biomarkers in clinics. Lastly, as ADRD treatments advance, increasing numbers of clinical studies and trials are poised to screen and recruit participants using blood-based biomarkers as a risk-stratification tool, particularly in primary and secondary prevention trials to slow cognitive decline. For such studies, our findings stress the importance of simultaneously including the *APOE4* allele genotyping as a standard workflow given *APOE4*’s compounding effects in individuals with higher levels of blood-based neurodegenerative markers and association with an increased rate of cognitive decline and, by extension, risk of developing dementias.

## Notes

Supplement 1. **eFigure 1.** Flow diagram indicating participant inclusion and flow **eFigure 2.** Box plots of serum neurodegenerative biomarkers stratified by race and *APOE4* carrier status **eFigure 3.** An alternative way of presentation: longitudinal cognitive decline in the lower and upper tertiles of serum neurodegenerative biomarkers among *APOE4* carriers vs noncarriers **eTable 1.** Distribution of *APOE4* genotypes in the total sample ( N= 1038) **eTable 2.** Associations of concentrations of biomarkers with annual rate of cognitive decline, stratified by *APOE4* carriers status **eTable 3.** Sensitivity analyses on subset of cognitively unimpaired sample with baseline MCI and AD cases excluded: associations of concentrations of biomarkers with annual rate of cognitive decline, stratified by *APOE4* carriers status **eTable 4.** Total and stratified samples: 3-way and 2-way interaction models **eTable 5.** Spearman correlation coefficients of the biomarkers, cross-tabulations of biomarker tertiles, distributions of different combinations of biomarker tertiles, and total and stratified models examining combined biomarker tertiles

Supplement 2.
Data Sharing Statement
