# Error in Figure Key

**DOI:** 10.1001/jamanetworkopen.2025.58120

**Published:** 2026-01-16

In the Original Investigation titled “*APOE4*, Blood Neurodegenerative Biomarkers, and Cognitive Decline in Community-Dwelling Older Adults,”^1^ published May 7, 2025, the labels for *APOE4* carriers and noncarriers were reversed in Figure 1. The article has been corrected.^1^
