# Supplementary material for: APOE4, Blood Neurodegenerative Biomarkers, and Cognitive Decline in Community-Dwelling Older Adults
Source: JAMA Netw Open. 2025 May 7;8(5):e258903. doi: 10.1001/jamanetworkopen.2025.8903 (PMC12059971; doi:10.1001/jamanetworkopen.2025.8903)
Supplement: Supplement 1. — eFigure 1. Flow diagram indicating participant inclusion and flow eFigure 2. Box plots of serum neurodegenerative biomarkers stratified by race and APOE4 carrier status eFigure 3. An alternative way of presentation: longitudinal cognitive decline in the lower and upper tertiles of serum neurodegenerative biomarkers among APOE4 carriers vs noncarriers eTable 1. Distribution of APOE4 genotypes in the total sample ( N= 1038) eTable 2. Associations of concentrations of biomarkers with annual rate of cognitive decline, stratified by APOE4 carriers status eTable 3. Sensitivity analyses on subset of cognitively unimpaired sample with baseline MCI and AD cases excluded: associations of concentrations of biomarkers with annual rate of cognitive decline, stratified by APOE4 carriers status eTable 4. Total and stratified samples: 3-way and 2-way interaction models eTable 5. Spearman correlation coefficients of the biomarkers, cross-tabulations of biomarker tertiles, distributions of different combinations of biomarker tertiles, and total and stratified models examining combined biomarker tertiles [file jamanetwopen-e258903-s001.pdf]

Supplemental Online Content

Ng TKS, Beck T, Boyle P, et al. *APOE4*, blood neurodegenerative biomarkers, and cognitive decline in community-dwelling older adults. *JAMA Netw Open*. 2025;8(5):e258903. doi:10.1001/jamanetworkopen.2025.8903

eFigure 1. Flow diagram indicating participant inclusion and flow

eFigure 2. Box plots of serum neurodegenerative biomarkers stratified by race and *APOE4* carrier status

eFigure 3. An alternative way of presentation: longitudinal cognitive decline in the lower and upper tertiles of serum neurodegenerative biomarkers among *APOE4* carriers vs noncarriers

eTable 1. Distribution of *APOE4* genotypes in the total sample (N= 1038)

eTable 2. Associations of concentrations of biomarkers with annual rate of cognitive decline, stratified by *APOE4* carriers status

eTable 3. Sensitivity analyses on subset of cognitively unimpaired sample with baseline MCI and AD cases excluded: associations of concentrations of biomarkers with annual rate of cognitive decline, stratified by *APOE4* carriers status

eTable 4. Total and stratified samples: 3-way and 2-way interaction models

eTable 5. Spearman correlation coefficients of the biomarkers, cross-tabulations of biomarker tertiles, distributions of different combinations of biomarker tertiles, and total and stratified models examining combined biomarker tertiles

This supplemental material has been provided by the authors to give readers additional information about their work.

© 2025 Ng TKS et al. *JAMA Network Open*.

Supplementary Figure 1: Flowchart of CHAP study participants for Biomarker Assay Analysis

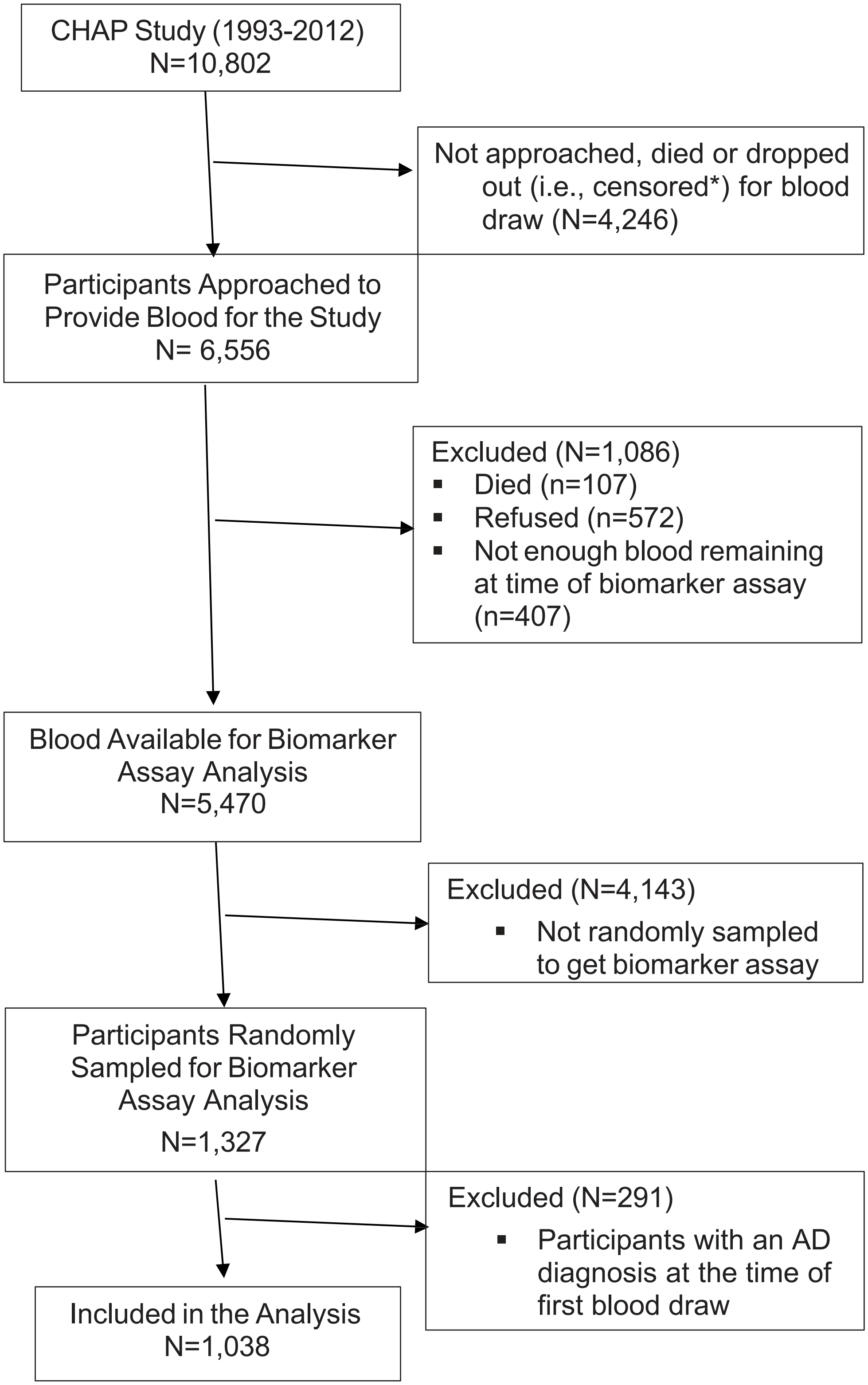

© 2025 Ng TKS et al. *JAMA Network Open*.

Note: *Censored includes death or lost to follow-up.
It is worth noting that not all 1038 participants included in the analyses were from a single wave of this study; As detailed in the methods section, there were participants who had not consented to blood sample collection in the fifth cycle, the first cycle of CHAP where we started blood sampling, but consented and provided blood samples in the subsequent cycles. Hence, 1,038 non-demented participants across the study waves fulfilled all the criteria and were included in this study.

© 2025 Ng TKS et al. *JAMA Network Open*.

Suppl Figure 2. Boxplots of Serum Neurodegenerative Biomarkers Stratified by Race and APOE4 carrier status

Legend: Raw levels of serum biomarkers by the APOE4 carrier status and Black and White participants.

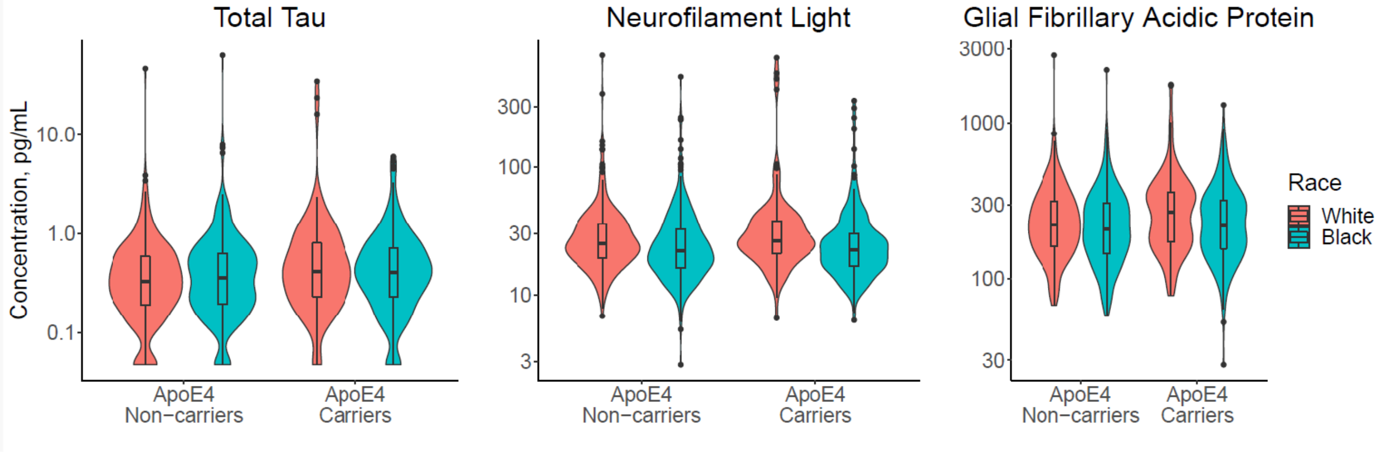

Suppl Figure 3. An alternative way of presentation: Longitudinal Cognitive Decline in the Lower and Upper Tertiles of Serum Neurodegenerative Biomarkers among APOE4 Carriers VS non-carriers

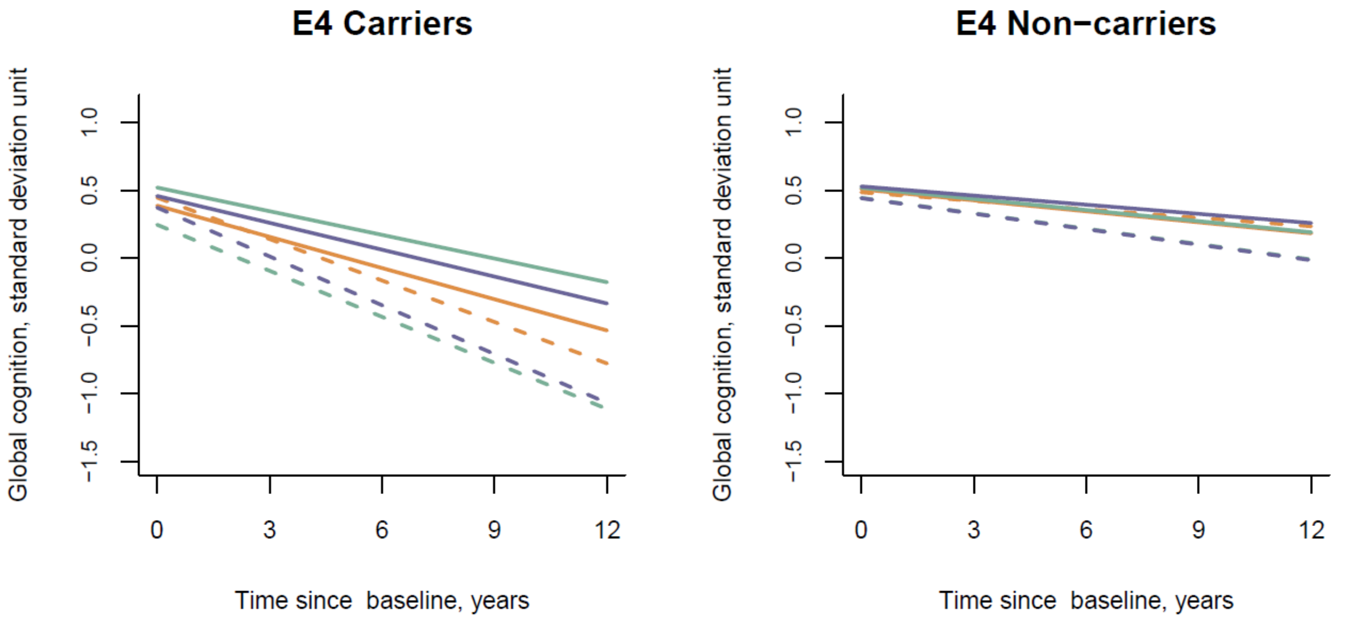

Note: Tau = orange; NFL = green; GFAP= purple;

Lower tertile = solid line; Upper tertile = dashed line

© 2025 Ng TKS et al. *JAMA Network Open*.

Supplementary Table 1. Distribution of APOE 4 Genotypes in the Total Sample (N=1,038)

genotype	Frequency	Percent	Cumulative Frequency	Cumulative Percent
ε2 ε2	5	0.48	5	0.48
ε2 ε3	149	14.35	154	14.84
ε2 ε4	46	4.43	200	19.27
ε3 ε3	541	52.12	741	71.39
ε3 ε4	265	25.53	1006	96.92
ε4 ε4	32	3.08	1038	100.00

Note: Due to the low numbers of homogenous ε4 ε4 and ε2 ε2, we focused the analyses on APOE 4 carriers VS non-carriers.

© 2025 Ng TKS et al. *JAMA Network Open*.

Supplementary Table 2a. Associations of Concentrations of Total Tau with Annual Rate of Cognitive Decline, Stratified by APOE4 Carriers Status in non-demented biracial community-dwelling older adults

	Non-APOE4 Carriers<br>Estimate<br>(SD, p-value)<br>n=693	APOE4 Carriers<br>Estimate<br>(SD, p-value)<br>n=340
Total Tau		
Linear, pg/ml	0.0225 (0.0428,0.5993)	0.0793 (0.0586,0.1773)
Intercept	0.5419 (0.0437,0.0000)	0.5093 (0.0636,0.0000)
Time	-0.0304 (0.0082,**<0.001*****)	-0.1169 (0.0169,**<0.001***)**
Total Tau*Time	-0.0038 (0.0084,0.6536)	-0.0365 (0.0159,**0.02***)
Total Tau		
Tertile Groups		
Lower (< 0.26 pg/ml) (n=275;103)	Reference	Reference

© 2025 Ng TKS et al. *JAMA Network Open*.

Middle (0.26 – 0.56 pg/ml) (n=225;123)	-0.0013 (0.0415,0.9753)	0.0537 (0.0619,0.3860)
Upper (>0.56 pg/ml) (n=193;116)	-0.0224 (0.0441,0.6114)	0.0592 (0.0638,0.3541)
Intercept	0.5371 (0.0437,0.0000)	0.4401 (0.0713,0.0000)
Time	-0.0298 (0.0079, ,**<0.001*****)	-0.0918 (0.0186, ,**<0.001***)**
Lower *Time	Reference	Reference
Middle*Time	-0.0005 (0.0080,0.9526)	-0.0060 (0.0164,0.7118)
UPPER*Time	0.0062 (0.0089,0.4868)	-0.0252 (0.0176,0.1514)

NOTE: The linear mixed effects regression models were adjusted for age (centered at 75), education (centered at 12), female sex, African American race, BMI, chronic conditions, and included the interaction of these characteristics with time since baseline. *** indicates <0.001; **indicates <0.01; *indicates <0.05.

© 2025 Ng TKS et al. *JAMA Network Open*.

Supplementary Table 2b. Associations of Concentrations of Glial Fibrillary Acidic Protein with Annual Rate of Cognitive Decline, Stratified by APOE4 Carriers Status in non-demented biracial community-dwelling older adults

	Non-APOE4 Carriers Estimate (SD, p-value) n=693	APOE4 Carriers Estimate (SD, p-value) n=340
Glial Fibrillary Acidic Protein		
Linear, pg/ml	-0.1432 (0.0868,0.0995)	-0.1161 (0.1163,0.3191)
Intercept	0.8616 (0.2038,0.0000)	0.7560 (0.2843,0.0082)
Time	0.0263 (0.0400,0.5106)	0.1743 (0.0779,**0.0256***)
Glial Fibrillary Acidic Protein*Time	-0.0239 (0.0172,0.1657)	-0.1158 (0.0319,**<0.001***)**
Glial Fibrillary Acidic Protein		
Tertile Groups		

© 2025 Ng TKS et al. *JAMA Network Open*.

Lower (<180 pg/ml) (n=254;119)	Reference	Reference
Middle (180 – 300 pg/ml) (n=251;105)	-0.0326 (0.0423,0.4407)	-0.0146 (0.0625,0.8160)
Upper (>300 pg/ml) (n=187;118)	-0.0874 (0.0496,0.0783)	-0.0859 (0.0662,0.1953)
Intercept	0.5615 (0.0441,0.0000)	0.5207 (0.0708,0.0000)
Time	-0.0245 (0.0080,**0.0023**)**	-0.0808 (0.0187,**<0.001***)**
Lower *Time	Reference	Reference
Middle*Time	-0.0042 (0.0080,0.5952)	-0.0059 (0.0165,0.7227)
Upper *Time	-0.0156 (0.0102,0.1274)	-0.0540 (0.0180,**0.0028****)

NOTE: The linear mixed effects regression models were adjusted for age (centered at 75), education (centered at 12), female sex, African American race, BMI, chronic conditions, and included the interaction of these characteristics with time since baseline. *** indicates <0.001; **indicates <0.01; *indicates <0.05.

© 2025 Ng TKS et al. *JAMA Network Open*.

Supplementary Table 3a. Sensitivity Analyses on Subset of Cognitively Unimpaired Sample, i.e., baseline MCI and AD cases excluded: Associations of Concentrations of Total Tau with Annual Rate of Cognitive Decline, Stratified by APOE4 Cariers Status in non-demented biracial community-dwelling older adults

	Total Sample	Non-APOE4 Carriers<br>Estimate<br>(SD, p-value)<br>n=353	APOE4 Carriers<br>Estimate<br>(SD, p-value)<br>n=170
Total Tau			
Linear, pg/ml	0.0273 (0.0497,0.5834)	0.0207 (0.0503,0.6808)	0.0142 (0.0750,0.8503)
APOE 4 Status	0.0058 (0.0521,0.9115)	-	-
Intercept	0.6713 (0.0440,0.0000)	0.6621 (0.0493,0.0000)	0.6914 (0.0746,0.0000)
Time	-0.0104 (0.0102,0.3085)	-0.0034 (0.0099,0.7286)	-0.0868 (0.0220,0.0001)
Total Tau*Time	0.0006 (0.0115,0.9588)	0.0020 (0.0105,0.8496)	-0.0366 (0.0218,**0.0G40**)
Total Tau			

© 2025 Ng TKS et al. *JAMA Network Open*.

Tertile Groups			
Lower (Below 0.26 pg/ml)	Reference	Reference	Reference
Middle (0.26 – 0.56 pg/ml)	-0.0193 (0.0471,0.6827)	-0.0221 (0.0475,0.6423)	0.0215 (0.0700,0.7593)
Upper (Above 0.56 pg/ml)	-0.0316 (0.0504,0.5315)	-0.0333 (0.0510,0.5145)	0.0180 (0.0778,0.8171)
APOE 4 Status	-0.0033 (0.0616,0.9579)	-	-
Intercept	0.6727 (0.0442,0.0000)	0.6682 (0.0495,0.0000)	0.6705 (0.0877,0.0000)
Time	-0.0103 (0.0094,0.2711)	-0.0051 (0.0092,0.5792)	-0.0503 (0.0245,0.0411)
LOWER *Time	Reference	Reference	Reference
MIDDLE*Time	-0.0011 (0.0103,0.9167)	-0.0005 (0.0094,0.9556)	-0.0127 (0.0187,0.4965)
UPPER*Time	0.0030 (0.0116,0.7998)	0.0041 (0.0107,0.7050)	-0.0457 (0.0233,**0.0507**)

NOTE: The linear mixed effects regression models were adjusted for age (centered at 75), education (centered at 12), female sex, African American race, BMI, chronic conditions, and included the interaction of these characteristics with time since baseline. *** indicates <0.001; **indicates <0.01; *indicates <0.05.

© 2025 Ng TKS et al. *JAMA Network Open*.

Supplementary Table 3b. Sensitivity Analyses on Subset of Cognitively Unimpaired Sample, i.e., baseline MCI and AD cases excluded: Associations of Concentrations of Neurofilament Light with Annual Rate of Cognitive Decline, Stratified by APOE4 Carriers Status in non-demented biracial community-dwelling older adults

	Total Sample	Non-APOE4 Carriers<br>Estimate<br>(SD, p-value)<br>n=353	APOE4 Carriers<br>Estimate<br>(SD, p-value)<br>n=170
Neurofilament Light			
Linear, pg/ml	-0.1217 (0.0913,0.1834)	-0.1240 (0.0956,0.1956)	-0.1385 (0.1287,0.2834)
APOE 4 Status	0.0385 (0.2014,0.8483)	-	-
Intercept	0.8202 (0.1264,0.0000)	0.8143 (0.1309,0.0000)	0.8768 (0.1907,0.0000)
Time	0.0222 (0.0301,0.4623)	0.0199 (0.0284,0.4825)	-0.0097 (0.0519,0.8524)
Neurofilament Light*Time	-0.0255 (0.0224,0.2553)	-0.0193 (0.0214,0.3686)	-0.0449 (0.0354,0.2049)
Neurofilament Light *Tertile Groups*			

© 2025 Ng TKS et al. *JAMA Network Open*.

LOWER (Below 20.5 pg/ml)	Reference	Reference	Reference
MIDDLE (20.5 – 31.5 pg/ml)	-0.0206 (0.0495,0.6778)	-0.0204 (0.0515,0.6929)	0.0375 (0.0677,0.5808)
UPPER (Above 31.5 pg/ml)	-0.0599 (0.0554,0.2794)	-0.0576 (0.0581,0.3220)	-0.1477 (0.0813,0.0711)
APOE 4 Status	0.0142 (0.0538,0.7926)	-	-
Intercept	0.6729 (0.0431,0.0000)	0.6669 (0.0480,0.0000)	0.7025 (0.0781,0.0000)
Time	-0.0101 (0.0090,0.2650)	-0.0046 (0.0089,0.6029)	-0.0531 (0.0211,0.0122)
LOWER *Time	Reference	Reference	Reference
MIDDLE*Time	0.0003 (0.0109,0.9809)	0.0028 (0.0104,0.7884)	-0.0329 (0.0191,**0.0857**)
UPPER*Time	-0.0127 (0.0127,0.3160)	-0.0075 (0.0121,0.5349)	-0.0438 (0.0231,**0.058G**)

NOTE: The linear mixed effects regression models were adjusted for age (centered at 75), education (centered at 12), female sex, African American race, BMI, chronic conditions, and included the interaction of these characteristics with time since baseline. *** indicates <0.001; **indicates <0.01; *indicates <0.05.

© 2025 Ng TKS et al. *JAMA Network Open*.

Supplementary Table 3c. Sensitivity Analyses on Subset of Cognitively Unimpaired Sample, i.e., baseline MCI and AD cases excluded: Associations of Concentrations of Glial Fibrillary Acidic Protein with Annual Rate of Cognitive Decline, Stratified by APOE4 Carriers Status in non-demented biracial community-dwelling older adults

	Total Sample	Non-APOE4 Carriers<br>Estimate<br>(SD, p-value)<br>n=352	APOE4 Carriers<br>Estimate<br>(SD, p-value)<br>n=170
Glial Fibrillary Acidic Protein			
Linear, pg/ml	-0.1224 (0.1034,0.2373)	-0.1343 (0.1077,0.2132)	-0.0832 (0.1495,0.5786)
APOE 4 Status	-0.1060 (0.3877,0.7847)	-	-
Intercept	0.9383 (0.2398,0.0001)	0.9591 (0.2494,0.0001)	0.8818 (0.3637,0.0165)
Time	-0.0044 (0.0530,0.9340)	-0.0072 (0.0510,0.8881)	0.2048 (0.1038,0.0493)
Glial Fibrillary Acidic Protein*Time	-0.0025 (0.0230,0.9135)	0.0012 (0.0222,0.9584)	-0.1160 (0.0429,**0.0072**)

© 2025 Ng TKS et al. *JAMA Network Open*.

Glial Fibrillary Acidic Protein			
Tertile Groups			
LOWER (Below 180 pg/ml)	Reference	Reference	Reference
MIDDLE (180 – 300 pg/ml)	-0.0568 (0.0465,0.2220)	-0.0612 (0.0475,0.1983)	-0.1327 (0.0681,0.0532)
UPPER (Above 300 pg/ml)	-0.0697 (0.0556,0.2110)	-0.0757 (0.0576,0.1896)	-0.0417 (0.0788,0.5976)
APOE 4 Status	0.0370 (0.0569,0.5154)	-	-
Intercept	0.6900 (0.0434,0.0000)	0.6879 (0.0486,0.0000)	0.7438 (0.0822,0.0000)
Time	-0.0123 (0.0091,0.1798)	-0.0069 (0.0092,0.4547)	-0.0447 (0.0218,0.0409)
LOWER *Time	Reference	Reference	Reference
MIDDLE*Time	0.0083 (0.0099,0.3976)	0.0071 (0.0093,0.4480)	-0.0110 (0.0180,0.5427)
UPPER*Time	-0.0082 (0.0132,0.5334)	-0.0062 (0.0128,0.6262)	-0.0681 (0.0209,**0.0012**)

© 2025 Ng TKS et al. *JAMA Network Open*.

NOTE: The linear mixed effects regression models were adjusted for age (centered at 75), education (centered at 12), female sex, African American race, BMI, chronic conditions, and included the interaction of these characteristics with time since baseline. *** indicates <0.001; **indicates <0.01; *indicates <0.05.

© 2025 Ng TKS et al. *JAMA Network Open*.

Supplementary Table 4a. Total and stratified samples:3-way and 2-way interaction models with the interaction term “Log_{10} T-Tau levels * APOE4*time” and association with MMSE scores, and stratified by APOE4 carrier status, respectively.

	Total Sample	non- APOE4 carriers	APOE4 carriers
	Estimate (SD, p-value)	Estimate (SD, p-value)	Estimate (SD, p-value)
n	1033	693	340
Log_{10} T-Tau, pg/ml	0.0199 (0.0332,0.5498)	0.0157 (0.0320,0.6233)	0.0616 (0.0501,0.2197)
APOE4	-0.0160 (0.0340,0.6369)	-	-
Intercept	0.5179 (0.0304,0.0000)	0.5273 (0.0326,0.0000)	0.4717 (0.0544,0.0000)
Time	-0.0494 (0.0097,0.0000)	-0.0426 (0.0092,0.0000)	-0.1230 (0.0208,0.0000)
Log_{10} T-Tau *Time	-0.0100 (0.0107,0.3480)	-0.0102 (0.0092,0.2720)	-0.0349 (0.0196,**0.0756**)
Log_{10} T-Tau *APOE4	0.0284 (0.0561,0.6128)	-	-
APOE4*Time	-0.0586 (0.0117,0.0000)	-	-
Log_{10} T-Tau *APOE4*Time	-0.0230 (0.0186,0.2159)	-	-

© 2025 Ng TKS et al. *JAMA Network Open*.

NOTE: The linear mixed effects regression models were adjusted for age (centered at 75), education (centered at 12), female sex, African American race/ethnicity, BMI, hypertension, and diabetes, and included the interaction of these characteristics with time since baseline.

IL-6=interleukin-6; APOE=apolipoprotein E; *** indicates <0.0001; **indicates <0.01; *indicates <0.05.

© 2025 Ng TKS et al. *JAMA Network Open*.

Supplementary Table 4b. Total and stratified samples: 3-way and 2-way interaction models with the interaction term "T-Tau Tertiles*APOE4*time" and association with MMSE scores, and stratified by APOE4 carrier status, respectively.

	Total Sample	non- APOE4 carriers	APOE4 carriers
	Estimate (SD, p-value)	Estimate (SD, p-value)	Estimate (SD, p-value)
n	1033	693	340
T-Tau Lower Tertile	Reference	Reference	Reference
T-Tau Middle Tertile	0.0166 (0.0323,0.6071)	0.0149 (0.0311,0.6312)	0.0375 (0.0529,0.4791)
T-Tau Upper Tertile	0.0066 (0.0342,0.8467)	0.0035 (0.0331,0.9169)	0.0532 (0.0546,0.3299)
APOE4	-0.0412 (0.0418,0.3248)	-	-
Intercept	0.5009 (0.0304,0.0000)	0.5137 (0.0326,0.0000)	0.4172 (0.0608,0.0000)
Time	-0.0430 (0.0093,0.0000)	-0.0363 (0.0089,0.0000)	-0.0967 (0.0228,0.0000)
T-Tau Lower Tertile*Time	Reference	Reference	Reference
T-Tau Middle Tertile*Time	0.0027 (0.0101,0.7862)	0.0023 (0.0088,0.7915)	-0.0003 (0.0199,0.9876)
T-Tau Upper Tertile*Time	-0.0070 (0.0111,0.5286)	-0.0067 (0.0097,0.4871)	-0.0359 (0.0212,**0.0G16**)
T-Tau Lower Tertile*APOE4	Reference	Reference	Reference

© 2025 Ng TKS et al. *JAMA Network Open*.

T-Tau Middle Tertile*APOE4	0.0061 (0.0584,0.9168)	-	-
T-Tau Upper Tertile*APOE4	0.0389 (0.0600,0.5169)	-	-
APOE4*Time	-0.0405 (0.0130,0.0019)	-	-
T-Tau Lower Tertile*APOE4*Time	Reference	Reference	Reference
T-Tau Middle Tertile*APOE4*Time	-0.0006 (0.0187,0.9724)	-	-
T-Tau Upper Tertile*APOE4*Time	-0.0294 (0.0199,0.1388)	-	-

NOTE: The linear mixed effects regression models were adjusted for age (centered at 75), education (centered at 12), female sex, African American race/ethnicity, BMI, hypertension, and diabetes, and included the interaction of these characteristics with time since baseline.

IL-6=interleukin-6; APOE=apolipoprotein E; *** indicates <0.0001; **indicates <0.01; *indicates <0.05.

© 2025 Ng TKS et al. *JAMA Network Open*.

Supplementary Table 4c. Total and stratified samples: 3-way and 2-way interaction models with the interaction term "Log_{10} NfL levels * APOE4*time" and association with MMSE scores, and stratified by APOE4 carrier status, respectively.

	Total Sample<br>Estimate (SD, p-value)	non- APOE4 carriers<br>Estimate (SD, p-value)	APOE4 carriers<br>Estimate (SD, p-value)
n	1033	693	340
Log_{10} NfL, pg/ml	-0.0558 (0.0582,0.3377)	-0.0589 (0.0578,0.3090)	-0.2100 (0.0834,0.0123)
APOE4	0.2033 (0.1279,0.1123)	-	-
Intercept	0.5819 (0.0827,0.0000)	0.5981 (0.0822,0.0000)	0.7433 (0.1271,0.0000)
Time	0.0080 (0.0274,0.7692)	0.0045 (0.0245,0.8551)	0.0103 (0.0489,0.8329)
Log_{10} NfL *Time	-0.0393 (0.0196,0.0455)	-0.0316 (0.0177,0.0735)	-0.0849 (0.0321,**0.0083****)
Log_{10} NfL *APOE4	-0.1590 (0.0894,0.0757)	-	-
APOE4*Time	-0.0027 (0.0426,0.9494)	-	-
Log_{10} NfL *APOE4*Time	-0.0323 (0.0301,0.2829)	-	-

© 2025 Ng TKS et al. *JAMA Network Open*.

NOTE: The linear mixed effects regression models were adjusted for age (centered at 75), education (centered at 12), female sex, African American race/ethnicity, BMI, hypertension, and diabetes, and included the interaction of these characteristics with time since baseline.

IL-6=interleukin-6; APOE=apolipoprotein E; *** indicates <0.0001; **indicates <0.01; *indicates <0.05.

© 2025 Ng TKS et al. *JAMA Network Open*.

Supplementary Table 4d. Total and stratified samples:3-way and 2-way interaction models with the interaction term “NfL Tertiles* APOE4*time” and association with MMSE scores, and stratified by APOE4 carrier status, respectively.

	Total Sample	non- APOE4 carriers	APOE4 carriers
	Estimate (SD, p-value)	Estimate (SD, p-value)	Estimate (SD, p-value)
n	1033	693	340
NfL Lower Tertile	Reference	Reference	Reference
NfL Middle Tertile	0.0147 (0.0334,0.6593)	0.0139 (0.0329,0.6731)	-0.0097 (0.0515,0.8503)
NfL Upper Tertile	-0.0289 (0.0365,0.4285)	-0.0278 (0.0366,0.4479)	-0.1910 (0.0589,0.0013)
APOE4	0.0318 (0.0391,0.4160)	-	-
Intercept	0.5090 (0.0300,0.0000)	0.5197 (0.0323,0.0000)	0.5124 (0.0579,0.0000)
Time	-0.0414 (0.0092,0.0000)	-0.0359 (0.0087,0.0000)	-0.0718 (0.0216,0.0009)
NfL Lower Tertile*Time	Reference	Reference	Reference
NfL Middle Tertile*Time	0.0005 (0.0105,0.9637)	0.0037 (0.0093,0.6937)	-0.0557 (0.0199,**0.0054**)**

© 2025 Ng TKS et al. *JAMA Network Open*.

NfL Upper Tertile*Time	-0.0240 (0.0118,0.0430)	-0.0170 (0.0107,0.1106)	-0.0767 (0.0228,**0.0008*****)
NfL Lower Tertile*APOE4	Reference	Reference	Reference
NfL Middle Tertile*APOE4	-0.0269 (0.0563,0.6328)	-	-
NfL Upper Tertile*APOE4	-0.1605 (0.0599,0.0075)	-	-
APOE4*Time	-0.0220 (0.0126,0.0801)	-	-
NfL Lower Tertile*APOE4*Time	Reference	Reference	Reference
NfL Middle Tertile*APOE4*Time	-0.0450 (0.0183,0.0139)	-	-
NfL Upper Tertile*APOE4*Time	-0.0396 (0.0200,0.0480)	-	-

NOTE: The linear mixed effects regression models were adjusted for age (centered at 75), education (centered at 12), female sex, African American race/ethnicity, BMI, hypertension, and diabetes, and included the interaction of these characteristics with time since baseline.

IL-6=interleukin-6; APOE=apolipoprotein E; *** indicates <0.0001; **indicates <0.01; *indicates <0.05.

© 2025 Ng TKS et al. *JAMA Network Open*.

Supplementary Table 4e. Total and stratified samples: 3-way and 2-way interaction models with the interaction term "Log_{10} GFAP levels * APOE4*time" and association with MMSE scores, and stratified by APOE4 carrier status, respectively.

	Total Sample	non- APOE4 carriers	APOE4 carriers
	Estimate (SD, p-value)	Estimate (SD, p-value)	Estimate (SD, p-value)
n	1033	693	340
Log_{10} GFAP, pg/ml	-0.0673 (0.0656,0.3051)	-0.0778 (0.0652,0.2330)	-0.1525 (0.0998,0.1276)
APOE4	0.2585 (0.2410,0.2838)	-	-
Intercept	0.6642 (0.1538,0.0000)	0.6989 (0.1529,0.0000)	0.8128 (0.2440,0.0010)
Time	0.0427 (0.0498,0.3914)	0.0470 (0.0441,0.2867)	0.0901 (0.0948,0.3422)
Log_{10} GFAP *Time	-0.0377 (0.0213,0.0774)	-0.0366 (0.0189,**0.0528**)	-0.0835 (0.0388,**0.0320***)
Log_{10} GFAP *APOE4	-0.1180 (0.1018,0.2466)	-	-
APOE4*Time	0.0346 (0.0808,0.6682)	-	-
Log_{10} GFAP *APOE4*Time	-0.0346 (0.0342,0.3124)	-	-

© 2025 Ng TKS et al. *JAMA Network Open*.

NOTE: The linear mixed effects regression models were adjusted for age (centered at 75), education (centered at 12), female sex, African American race/ethnicity, BMI, hypertension, and diabetes, and included the interaction of these characteristics with time since baseline.

IL-6=interleukin-6; APOE=apolipoprotein E; *** indicates <0.0001; **indicates <0.01; *indicates <0.05.

© 2025 Ng TKS et al. *JAMA Network Open*.

Supplementary Table 4f. Total and stratified samples: 3-way and 2-way interaction models with the interaction term "GFAP Tertiles* APOE4*time" and association with MMSE scores, and stratified by APOE4 carrier status, respectively.

	Total Sample<br>Estimate (SD, p-value)	non- APOE4 carriers<br>Estimate (SD, p-value)	APOE4 carriers<br>Estimate (SD, p-value)
n	1033	693	340
GFAP Lower Tertile	Reference	Reference	Reference
GFAP Middle Tertile	-0.0232 (0.0325,0.4752)	-0.0271 (0.0317,0.3928)	0.0050 (0.0534,0.9251)
GFAP Upper Tertile	-0.0261 (0.0377,0.4892)	-0.0305 (0.0373,0.4145)	-0.0908 (0.0566,0.1097)
APOE4	-0.0041 (0.0402,0.9190)	-	-
Intercept	0.5246 (0.0309,0.0000)	0.5364 (0.0330,0.0000)	0.4849 (0.0604,0.0000)
Time	-0.0387 (0.0096,0.0001)	-0.0319 (0.0090,0.0004)	-0.0980 (0.0230,0.0000)
GFAP Lower Tertile*Time	Reference	Reference	Reference
GFAP Middle Tertile*Time	-0.0024 (0.0102,0.8144)	-0.0040 (0.0088,0.6521)	0.0054 (0.0204,0.7922)
GFAP Upper Tertile*Time	-0.0238 (0.0124,0.0544)	-0.0243 (0.0110,**0.0272***)	-0.0324 (0.0221,0.1425)

© 2025 Ng TKS et al. *JAMA Network Open*.

GFAP Lower Tertile*APOE4	Reference	Reference	Reference
GFAP Middle Tertile*APOE4	0.0227 (0.0581,0.6960)	-	-
GFAP Upper Tertile*APOE4	-0.0773 (0.0592,0.1919)	-	-
APOE4*Time	-0.0466 (0.0130,0.0004)	-	-
GFAP Lower Tertile*APOE4*Time	Reference	Reference	Reference
GFAP Middle Tertile*APOE4*Time	0.0046 (0.0188,0.8060)	-	-
GFAP Upper Tertile*APOE4*Time	-0.0061 (0.0198,0.7585)	-	-

NOTE: The linear mixed effects regression models were adjusted for age (centered at 75), education (centered at 12), female sex, African American race/ethnicity, BMI, hypertension, and diabetes, and included the interaction of these characteristics with time since baseline.

IL-6=interleukin-6; APOE=apolipoprotein E; *** indicates <0.0001; **indicates <0.01; *indicates <0.05.

© 2025 Ng TKS et al. *JAMA Network Open*.

Supplementary Table 4g. Total and stratified samples:3-way and 2-way interaction models with the interaction term "Log_{10} T-Tau levels * APOE4*time" and association with episodic memory, and stratified by APOE4 carrier status, respectively.

	Total Sample<br>Estimate (SD, p-value)	non- APOE4 carriers<br>Estimate (SD, p-value)	APOE4 carriers<br>Estimate (SD, p-value)
n	1033	693	340
Log_{10} T-Tau, pg/ml	0.0378 (0.0599,0.5280)	0.0399 (0.0614,0.5165)	0.1334 (0.0802,0.0971)
APOE4	0.0128 (0.0612,0.8348)	-	-
Intercept	0.4190 (0.0549,0.0000)	0.4255 (0.0628,0.0000)	0.4233 (0.0871,0.0000)
Time	-0.0174 (0.0101,0.0867)	-0.0112 (0.0100,0.2609)	-0.1058 (0.0215,0.0000)
Log_{10} T-Tau *Time	0.0011 (0.0112,0.9233)	0.0003 (0.0103,0.9738)	-0.0512 (0.0202,**0.0114***)
Log_{10} T-Tau *APOE4	0.0912 (0.1012,0.3676)	-	-
APOE4*Time	-0.0671 (0.0132,0.0000)	-	-
Log_{10} T-Tau *APOE4*Time	-0.0501 (0.0201,0.0128)	-	-

© 2025 Ng TKS et al. *JAMA Network Open*.

NOTE: The linear mixed effects regression models were adjusted for age (centered at 75), education (centered at 12), female sex, African American race/ethnicity, BMI, hypertension, and diabetes, and included the interaction of these characteristics with time since baseline.

IL-6=interleukin-6; APOE=apolipoprotein E; *** indicates <0.0001; **indicates <0.01; *indicates <0.05.

© 2025 Ng TKS et al. *JAMA Network Open*.

Supplementary Table 4h. Total and stratified samples:3-way and 2-way interaction models with the interaction term "T-Tau Tertiles*APOE4*time" and association with episodic memory, and stratified by APOE4 carrier status, respectively.

	Total Sample	non- APOE4 carriers	APOE4 carriers
	Estimate (SD, p-value)	Estimate (SD, p-value)	Estimate (SD, p-value)
n	1033	693	340
T-Tau Lower Tertile	Reference	Reference	Reference
T-Tau Middle Tertile	0.0195 (0.0585,0.7392)	0.0219 (0.0598,0.7147)	0.0917 (0.0848,0.2804)
T-Tau Upper Tertile	-0.0319 (0.0619,0.6065)	-0.0240 (0.0634,0.7052)	0.1208 (0.0875,0.1681)
APOE4	-0.0929 (0.0752,0.2172)	-	-
Intercept	0.4027 (0.0553,0.0000)	0.4057 (0.0630,0.0000)	0.2988 (0.0976,0.0024)
Time	-0.0198 (0.0096,0.0391)	-0.0129 (0.0096,0.1819)	-0.0676 (0.0234,0.0041)
T-Tau Lower Tertile*Time	Reference	Reference	Reference
T-Tau Middle Tertile*Time	-0.0036 (0.0107,0.7348)	-0.0053 (0.0098,0.5914)	-0.0122 (0.0208,0.5555)
T-Tau Upper Tertile*Time	0.0138 (0.0119,0.2492)	0.0124 (0.0111,0.2639)	-0.0404 (0.0223,**0.0708**)

© 2025 Ng TKS et al. *JAMA Network Open*.

T-Tau Lower Tertile*APOE4	Reference	Reference	Reference
T-Tau Middle Tertile*APOE4	0.0529 (0.1052,0.6152)	-	-
T-Tau Upper Tertile*APOE4	0.1663 (0.1080,0.1241)	-	-
APOE4*Time	-0.0279 (0.0140,0.0461)	-	-
T-Tau Lower Tertile*APOE4*Time	Reference	Reference	Reference
T-Tau Middle Tertile*APOE4*Time	-0.0026 (0.0203,0.8989)	-	-
T-Tau Upper Tertile*APOE4*Time	-0.0579 (0.0220,0.0087)	-	-

NOTE: The linear mixed effects regression models were adjusted for age (centered at 75), education (centered at 12), female sex, African American race/ethnicity, BMI, hypertension, and diabetes, and included the interaction of these characteristics with time since baseline.

IL-6=interleukin-6; APOE=apolipoprotein E; *** indicates <0.0001; **indicates <0.01; *indicates <0.05.

© 2025 Ng TKS et al. *JAMA Network Open*.

Supplementary Table 4i. Total and stratified samples: 3-way and 2-way interaction models with the interaction term "Log_{10} NfL levels * APOE4*time" and association with episodic memory, and stratified by APOE4 carrier status, respectively.

	Total Sample	non- APOE4 carriers	APOE4 carriers
	Estimate (SD, p-value)	Estimate (SD, p-value)	Estimate (SD, p-value)
n	1033	693	340
Log_{10} NfL, pg/ml	-0.1258 (0.1060,0.2356)	-0.1084 (0.1115,0.3312)	-0.2477 (0.1353,0.0681)
APOE4	0.1056 (0.2321,0.6493)	-	-
Intercept	0.5702 (0.1505,0.0002)	0.5521 (0.1582,0.0005)	0.7216 (0.2059,0.0005)
Time	0.0151 (0.0292,0.6046)	0.0195 (0.0277,0.4806)	0.0134 (0.0525,0.7982)
Log_{10} NfL *Time	-0.1258 (0.1060,0.2356)	-0.1084 (0.1115,0.3312)	-0.2477 (0.1353,**0.0681**)
Log_{10} NfL *APOE4	-0.0848 (0.1623,0.6014)	-	-
APOE4*Time	0.0165 (0.0484,0.7339)	-	-
Log_{10} NfL *APOE4*Time	-0.0440 (0.0348,0.2056)	-	-

NOTE: The linear mixed effects regression models were adjusted for age (centered at 75), education (centered at 12), female sex, African American race/ethnicity, BMI, hypertension, and diabetes, and included the interaction of these characteristics with time since

© 2025 Ng TKS et al. *JAMA Network Open*.

baseline.

© 2025 Ng TKS et al. *JAMA Network Open*.

IL-6=interleukin-6; APOE=apolipoprotein E; *** indicates <0.0001; **indicates <0.01; *indicates <0.05.

© 2025 Ng TKS et al. *JAMA Network Open*.

Supplementary Table 4j. Total and stratified samples:3-way and 2-way interaction models with the interaction term "NfL Tertiles* APOE4*time" and association with episodic memory, and stratified by APOE4 carrier status, respectively.

	Total Sample	non- APOE4 carriers	APOE4 carriers
	Estimate (SD, p-value)	Estimate (SD, p-value)	Estimate (SD, p-value)
n	1033	693	340
NfL Lower Tertile	Reference	Reference	Reference
NfL Middle Tertile	-0.0336 (0.0609,0.5815)	-0.0294 (0.0632,0.6426)	-0.0358 (0.0833,0.6673)
NfL Upper Tertile	-0.0809 (0.0666,0.2246)	-0.0653 (0.0703,0.3531)	-0.2397 (0.0957,0.0127)
APOE4	0.0210 (0.0710,0.7671)	-	-
Intercept	0.4273 (0.0549,0.0000)	0.4264 (0.0623,0.0000)	0.4627 (0.0939,0.0000)
Time	-0.0213 (0.0093,0.0222)	-0.0146 (0.0093,0.1153)	-0.0548 (0.0217,0.0117)
NfL Lower Tertile*Time	Reference	Reference	Reference
NfL Middle Tertile*Time	0.0133 (0.0111,0.2302)	0.0146 (0.0104,0.1615)	-0.0561 (0.0206,**0.0066***)

© 2025 Ng TKS et al. *JAMA Network Open*.

NfL Upper Tertile*Time	-0.0110 (0.0129,0.3908)	-0.0077 (0.0123,0.5324)	-0.0619 (0.0241,**0.0106***)
NfL Lower Tertile*APOE4	Reference	Reference	Reference
NfL Middle Tertile*APOE4	0.0005 (0.1021,0.9960)	-	-
NfL Upper Tertile*APOE4	-0.1233 (0.1088,0.2572)	-	-
APOE4*Time	-0.0144 (0.0132,0.2770)	-	-
NfL Lower Tertile*APOE4*Time	Reference	Reference	Reference
NfL Middle Tertile*APOE4*Time	-0.0565 (0.0196,0.0040)	-	-
NfL Upper Tertile*APOE4*Time	-0.0463 (0.0227,0.0420)	-	-

NOTE: The linear mixed effects regression models were adjusted for age (centered at 75), education (centered at 12), female sex, African American race/ethnicity, BMI, hypertension, and diabetes, and included the interaction of these characteristics with time since baseline. IL-6=interleukin-6; APOE=apolipoprotein E; *** indicates <0.0001; **indicates <0.01; *indicates <0.05.

© 2025 Ng TKS et al. *JAMA Network Open*.

Supplementary Table 4k. Total and stratified samples:3-way and 2-way interaction models with the interaction term "Log_{10} GFAP levels * APOE4*time" and association with episodic memory, and stratified by APOE4 carrier status, respectively.

	Total Sample<br>Estimate (SD, p-value)	non- APOE4 carriers<br>Estimate (SD, p-value)	APOE4 carriers<br>Estimate (SD, p-value)
n	1033	693	340
Log_{10} GFAP, pg/ml	-0.1423 (0.1189,0.2317)	-0.1507 (0.1253,0.2292)	-0.0148 (0.1590,0.9257)
APOE4	-0.2581 (0.4344,0.5525)	-	-
Intercept	0.7292 (0.2787,0.0090)	0.7547 (0.2940,0.0105)	0.4062 (0.3886,0.2966)
Time	0.0213 (0.0517,0.6807)	0.0140 (0.0493,0.7756)	0.2756 (0.0983,0.0052)
Log_{10} GFAP *Time	-0.1423 (0.1189,0.2317)	-0.1507 (0.1253,0.2292)	-0.0148 (0.1590,0.9257)
Log_{10} GFAP *APOE4	0.1040 (0.1836,0.5710)	-	-
APOE4*Time	0.2174 (0.0893,0.0150)	-	-
Log_{10} GFAP *APOE4*Time	-0.1109 (0.0381,0.0036)	-	-

© 2025 Ng TKS et al. *JAMA Network Open*.

NOTE: The linear mixed effects regression models were adjusted for age (centered at 75), education (centered at 12), female sex, African American race/ethnicity, BMI, hypertension, and diabetes, and included the interaction of these characteristics with time since baseline.

IL-6=interleukin-6; APOE=apolipoprotein E; *** indicates <0.0001; **indicates <0.01; *indicates <0.05.

© 2025 Ng TKS et al. *JAMA Network Open*.

Supplementary Table 4l. Total and stratified samples:3-way and 2-way interaction models with the interaction term "GFAP Tertiles* APOE4*time" and association with episodic memory, and stratified by APOE4 carrier status, respectively.

	Total Sample	non- APOE4 carriers	APOE4 carriers
	Estimate (SD, p-value)	Estimate (SD, p-value)	Estimate (SD, p-value)
n	1033	693	340
GFAP Lower Tertile	Reference	Reference	Reference
GFAP Middle Tertile	-0.0367 (0.0589,0.5331)	-0.0457 (0.0609,0.4533)	0.0266 (0.0855,0.7561)
GFAP Upper Tertile	-0.0904 (0.0684,0.1867)	-0.0944 (0.0717,0.1883)	-0.0252 (0.0905,0.7806)
APOE4	-0.0531 (0.0725,0.4636)	-	-
Intercept	0.4360 (0.0561,0.0000)	0.4436 (0.0636,0.0000)	0.3799 (0.0969,0.0001)
Time	-0.0147 (0.0097,0.1304)	-0.0097 (0.0097,0.3179)	-0.0546 (0.0231,0.0184)
GFAP Lower Tertile*Time	Reference	Reference	Reference
GFAP Middle Tertile*Time	-0.0015 (0.0105,0.8847)	-0.0015 (0.0098,0.8746)	-0.0126 (0.0203,0.5360)

© 2025 Ng TKS et al. *JAMA Network Open*.

GFAP Upper Tertile*Time	-0.0134 (0.0136,0.3246)	-0.0105 (0.0130,0.4206)	-0.0747 (0.0224,**0.000G*****)
GFAP Lower Tertile*APOE4	Reference	Reference	Reference
GFAP Middle Tertile*APOE4	0.0601 (0.1048,0.5662)	-	-
GFAP Upper Tertile*APOE4	0.0672 (0.1069,0.5299)	-	-
APOE4*Time	-0.0224 (0.0136,0.1004)	-	-
GFAP Lower Tertile*APOE4*Time	Reference	Reference	Reference
GFAP Middle Tertile*APOE4*Time	-0.0149 (0.0200,0.4558)	-	-
GFAP Upper Tertile*APOE4*Time	-0.0553 (0.0221,0.0124)	-	-

NOTE: The linear mixed effects regression models were adjusted for age (centered at 75), education (centered at 12), female sex, African American race/ethnicity, BMI, hypertension, and diabetes, and included the interaction of these characteristics with time since baseline.

IL-6=interleukin-6; APOE=apolipoprotein E; *** indicates <0.0001; **indicates <0.01; *indicates <0.05.

© 2025 Ng TKS et al. *JAMA Network Open*.

Supplementary Table 4m. Total and stratified samples:3-way and 2-way interaction models with the interaction term "Log_{10} T-Tau levels * APOE4*time" and association with perceptual speed/executive function, and stratified by APOE4 carrier status, respectively.

	Total Sample	non- APOE4 carriers	APOE4 carriers
	Estimate (SD, p-value)	Estimate (SD, p-value)	Estimate (SD, p-value)
n	1033	693	340
Log_{10} T-Tau, pg/ml	-0.0017 (0.0616,0.9781)	-0.0091 (0.0600,0.8791)	-0.0176 (0.0911,0.8468)
APOE4	-0.1414 (0.0624,0.0236)	-	-
Intercept	0.8252 (0.0566,0.0000)	0.8238 (0.0615,0.0000)	0.6936 (0.0988,0.0000)
Time	-0.0554 (0.0086,0.0000)	-0.0487 (0.0092,0.0000)	-0.1030 (0.0162,0.0000)
Log_{10} T-Tau *Time	0.0020 (0.0094,0.8316)	0.0016 (0.0094,0.8636)	-0.0111 (0.0150,0.4604)
Log_{10} T-Tau *APOE4	-0.0530 (0.1037,0.6093)	-	-
APOE4*Time	-0.0293 (0.0109,0.0072)	-	-
Log_{10} T-Tau *APOE4*Time	-0.0126 (0.0168,0.4519)	-	-

© 2025 Ng TKS et al. *JAMA Network Open*.

NOTE: The linear mixed effects regression models were adjusted for age (centered at 75), education (centered at 12), female sex, African American race/ethnicity, BMI, hypertension, and diabetes, and included the interaction of these characteristics with time since baseline.

IL-6=interleukin-6; APOE=apolipoprotein E; *** indicates <0.0001; **indicates <0.01; *indicates <0.05.

© 2025 Ng TKS et al. *JAMA Network Open*.

Supplementary Table 4n. Total and stratified samples:3-way and 2-way interaction models with the interaction term "T-Tau Tertiles*APOE4*time" and association with perceptual speed/executive function, and stratified by APOE4 carrier status, respectively.

	Total Sample	non- APOE4 carriers	APOE4 carriers
	Estimate (SD, p-value)	Estimate (SD, p-value)	Estimate (SD, p-value)
n	1033	693	340
T-Tau Lower Tertile	Reference	Reference	Reference
T-Tau Middle Tertile	-0.0460 (0.0601,0.4449)	-0.0459 (0.0583,0.4319)	-0.0110 (0.0958,0.9082)
T-Tau Upper Tertile	-0.0368 (0.0636,0.5627)	-0.0446 (0.0619,0.4721)	-0.0412 (0.0989,0.6773)
APOE4	-0.1174 (0.0772,0.1287)	-	-
Intercept	0.8501 (0.0570,0.0000)	0.8543 (0.0617,0.0000)	0.7177 (0.1108,0.0000)
Time	-0.0585 (0.0083,0.0000)	-0.0515 (0.0089,0.0000)	-0.1041 (0.0178,0.0000)
T-Tau Lower Tertile*Time	Reference	Reference	Reference
T-Tau Middle Tertile*Time	-0.0016 (0.0091,0.8586)	-0.0018 (0.0090,0.8430)	0.0079 (0.0156,0.6110)
T-Tau Upper Tertile*Time	0.0108 (0.0101,0.2873)	0.0103 (0.0100,0.3039)	0.0080 (0.0168,0.6349)
T-Tau Lower Tertile*APOE4	Reference	Reference	Reference

© 2025 Ng TKS et al. *JAMA Network Open*.

T-Tau Middle Tertile*APOE4	0.0206 (0.1077,0.8480)	-	-
T-Tau Upper Tertile*APOE4	-0.0217 (0.1106,0.8444)	-	-
APOE4*Time	-0.0260 (0.0118,0.0280)	-	-
T-Tau Lower Tertile*APOE4*Time	Reference	Reference	Reference
T-Tau Middle Tertile*APOE4*Time	0.0104 (0.0171,0.5436)	-	-
T-Tau Upper Tertile*APOE4*Time	-0.0064 (0.0185,0.7283)	-	-

NOTE: The linear mixed effects regression models were adjusted for age (centered at 75), education (centered at 12), female sex, African American race/ethnicity, BMI, hypertension, and diabetes, and included the interaction of these characteristics with time since baseline.

IL-6=interleukin-6; APOE=apolipoprotein E; *** indicates <0.0001; **indicates <0.01; *indicates <0.05.

© 2025 Ng TKS et al. *JAMA Network Open*.

Supplementary Table 4o. Total and stratified samples: 3-way and 2-way interaction models with the interaction term "Log_{10} NfL levels * APOE4*time" and association with perceptual speed/executive function, and stratified by APOE4 carrier status, respectively.

	Total Sample	non- APOE4 carriers	APOE4 carriers
	Estimate (SD, p-value)	Estimate (SD, p-value)	Estimate (SD, p-value)
n	1033	693	340
Log_{10} NfL, pg/ml	-0.1319 (0.1072,0.2189)	-0.0985 (0.1076,0.3602)	-0.4939 (0.1502,0.0011)
APOE4	0.3596 (0.2352,0.1266)	-	-
Intercept	1.0010 (0.1528,0.0000)	0.9599 (0.1534,0.0000)	1.3948 (0.2294,0.0000)
Time	-0.0318 (0.0247,0.1992)	-0.0332 (0.0252,0.1887)	-0.0495 (0.0384,0.1983)
Log_{10} NfL *Time	-0.0186 (0.0179,0.2991)	-0.0124 (0.0183,0.4970)	-0.0351 (0.0257,0.1729)
Log_{10} NfL *APOE4	-0.3345 (0.1642,0.0419)	-	-
APOE4*Time	-0.0084 (0.0395,0.8322)	-	-
Log_{10} NfL *APOE4*Time	-0.0107 (0.0283,0.7039)	-	-

© 2025 Ng TKS et al. *JAMA Network Open*.

NOTE: The linear mixed effects regression models were adjusted for age (centered at 75), education (centered at 12), female sex, African American race/ethnicity, BMI, hypertension, and diabetes, and included the interaction of these characteristics with time since baseline.

IL-6=interleukin-6; APOE=apolipoprotein E; *** indicates <0.0001; **indicates <0.01; *indicates <0.05.

© 2025 Ng TKS et al. *JAMA Network Open*.

Supplementary Table 4p. Total and stratified samples:3-way and 2-way interaction models with the interaction term “NfL Tertiles* APOE4*time” and association with perceptual speed/executive function, and stratified by APOE4 carrier status, respectively.

	Total Sample	non- APOE4 carriers	APOE4 carriers
	Estimate (SD, p-value)	Estimate (SD, p-value)	Estimate (SD, p-value)
n	1033	693	340
NfL Lower Tertile	Reference	Reference	Reference
NfL Middle Tertile	0.0005 (0.0621,0.9931)	0.0103 (0.0616,0.8674)	-0.1384 (0.0931,0.1381)
NfL Upper Tertile	-0.1240 (0.0676,0.0670)	-0.1006 (0.0683,0.1410)	-0.4303 (0.1063,0.0001)
APOE4	0.0048 (0.0724,0.9475)	-	-
Intercept	0.8501 (0.0565,0.0000)	0.8431 (0.0613,0.0000)	0.8763 (0.1054,0.0000)
Time	-0.0523 (0.0081,0.0000)	-0.0466 (0.0087,0.0000)	-0.0903 (0.0166,0.0000)
NfL Lower Tertile*Time	Reference	Reference	Reference
NfL Middle Tertile*Time	-0.0065 (0.0094,0.4898)	-0.0035 (0.0094,0.7086)	-0.0212 (0.0157,0.1755)
NfL Upper Tertile*Time	-0.0163 (0.0110,0.1375)	-0.0128 (0.0112,0.2501)	-0.0148 (0.0184,0.4226)
NfL Lower Tertile*APOE4	Reference	Reference	Reference

© 2025 Ng TKS et al. *JAMA Network Open*.

NfL Middle Tertile*APOE4	-0.1285 (0.1039,0.2163)	-	-
NfL Upper Tertile*APOE4	-0.2691 (0.1102,0.0148)	-	-
APOE4*Time	-0.0225 (0.0112,0.0439)	-	-
NfL Lower Tertile*APOE4*Time	Reference	Reference	Reference
NfL Middle Tertile*APOE4*Time	-0.0064 (0.0166,0.6995)	-	-
NfL Upper Tertile*APOE4*Time	0.0070 (0.0190,0.7144)	-	-

NOTE: The linear mixed effects regression models were adjusted for age (centered at 75), education (centered at 12), female sex, African American race/ethnicity, BMI, hypertension, and diabetes, and included the interaction of these characteristics with time since baseline.

IL-6=interleukin-6; APOE=apolipoprotein E; *** indicates <0.0001; **indicates <0.01; *indicates <0.05.

© 2025 Ng TKS et al. *JAMA Network Open*.

Supplementary Table 4q. Total and stratified samples:3-way and 2-way interaction models with the interaction term "Log_{10} GFAP levels * APOE4*time" and association with perceptual speed/executive function, and stratified by APOE4 carrier status, respectively.

	Total Sample	non- APOE4 carriers	APOE4 carriers
	Estimate (SD, p-value)	Estimate (SD, p-value)	Estimate (SD, p-value)
n	1033	693	340
Log_{10} GFAP, pg/ml	-0.1719 (0.1215,0.1574)	-0.2010 (0.1216,0.0987)	-0.2903 (0.1795,0.1067)
APOE4	0.2505 (0.4421,0.5711)	-	-
Intercept	1.2243 (0.2853,0.0000)	1.2924 (0.2856,0.0000)	1.3937 (0.4386,0.0016)
Time	-0.0062 (0.0439,0.8887)	-0.0028 (0.0447,0.9496)	0.0374 (0.0755,0.6201)
Log_{10} GFAP *Time	-0.0218 (0.0189,0.2486)	-0.0203 (0.0192,0.2894)	-0.0571 (0.0310,**0.0663**)
Log_{10} GFAP *APOE4	-0.1525 (0.1867,0.4143)	-	-
APOE4*Time	0.0507 (0.0743,0.4952)	-	-
Log_{10} GFAP *APOE4*Time	-0.0309 (0.0316,0.3289)	-	-

© 2025 Ng TKS et al. *JAMA Network Open*.

NOTE: The linear mixed effects regression models were adjusted for age (centered at 75), education (centered at 12), female sex, African American race/ethnicity, BMI, hypertension, and diabetes, and included the interaction of these characteristics with time since baseline.

IL-6=interleukin-6; APOE=apolipoprotein E; *** indicates <0.0001; **indicates <0.01; *indicates <0.05.

© 2025 Ng TKS et al. *JAMA Network Open*.

Supplementary Table 4r. Total and stratified samples: 3-way and 2-way interaction models with the interaction term "GFAP Tertiles* APOE4*time" and association with perceptual speed/executive function, and stratified by APOE4 carrier status, respectively.

	Total Sample	non- APOE4 carriers	APOE4 carriers
	Estimate (SD, p-value)	Estimate (SD, p-value)	Estimate (SD, p-value)
n	1033	693	340
GFAP Lower Tertile	Reference	Reference	Reference
GFAP Middle Tertile	-0.0148 (0.0607,0.8080)	-0.0295 (0.0596,0.6212)	-0.0894 (0.0969,0.3574)
GFAP Upper Tertile	-0.1031 (0.0697,0.1395)	-0.1202 (0.0695,0.0839)	-0.1809 (0.1024,0.0783)
APOE4	-0.0567 (0.0742,0.4447)	-	-
Intercept	0.8562 (0.0578,0.0000)	0.8642 (0.0622,0.0000)	0.7990 (0.1097,0.0000)
Time	-0.0530 (0.0084,0.0000)	-0.0465 (0.0090,0.0000)	-0.0890 (0.0179,0.0000)
GFAP Lower Tertile*Time	Reference	Reference	Reference
GFAP Middle Tertile*Time	-0.0043 (0.0090,0.6337)	-0.0044 (0.0090,0.6271)	-0.0060 (0.0157,0.7017)
GFAP Upper Tertile*Time	-0.0110 (0.0115,0.3393)	-0.0101 (0.0116,0.3843)	-0.0237 (0.0173,0.1732)
GFAP Lower Tertile*APOE4	Reference	Reference	Reference

© 2025 Ng TKS et al. *JAMA Network Open*.

GFAP Middle Tertile*APOE4	-0.0831 (0.1071,0.4382)	-	-
GFAP Upper Tertile*APOE4	-0.0835 (0.1089,0.4433)	-	-
APOE4*Time	-0.0193 (0.0116,0.0960)	-	-
GFAP Lower Tertile*APOE4*Time	Reference	Reference	Reference
GFAP Middle Tertile*APOE4*Time	-0.0005 (0.0170,0.9762)	-	-
GFAP Upper Tertile*APOE4*Time	-0.0105 (0.0185,0.5723)	-	-

NOTE: The linear mixed effects regression models were adjusted for age (centered at 75), education (centered at 12), female sex, African American race/ethnicity, BMI, hypertension, and diabetes, and included the interaction of these characteristics with time since baseline.

IL-6=interleukin-6; APOE=apolipoprotein E; *** indicates <0.0001; **indicates <0.01; *indicates <0.05.

© 2025 Ng TKS et al. *JAMA Network Open*.

Supplementary Table 5a. Spearman Correlation Coefficients of the Biomarkers-Total Sample (N=1,041)

	T-Tau	NfL	GFAP
T-Tau	1	**0.22156*** **	**0.22560*** **
NfL		1	**0.53556*** **
GFAP			1

Note: *** indicates p<0.001.

Supplementary Table 5b. Spearman Correlation Coefficients of the Biomarkers-APOE4 Carrier Subgroup (n=342)

	T-Tau	NfL	GFAP
T-Tau	1	**0.27247*** **	**0.22G27*** **
NfL		1	**0.57463*** **
GFAP			1

Note: *** indicates p<0.001.

Supplementary Table 5c. Spearman Correlation Coefficients of the Biomarkers-non-APOE4 Carrier Subgroup (n=6G3)

	T-Tau	NfL	GFAP
T-Tau	1	**0.1G632*** **	**0.21626*** **
NfL		1	**0.51354*** **
GFAP			1

Note: *** indicates p<0.001.

© 2025 Ng TKS et al. *JAMA Network Open*.

Supplementary Table 5d. Cross-tabulations of the Biomarker Tertiles (N=1,034)

Biomarker Tertiles					Cumulative	Cumulative
T-Tau	NfL	GFAP	Frequency	Percent	Frequency	Percent
0	0	0	107	10.35	107	10.35
0	0	1	52	5.03	156	15.38
0	0	2	11	1.06	170	16.44
0	1	0	43	4.16	213	20.60
0	1	1	52	5.03	265	25.63
0	1	2	27	2.61	262	28.24
0	2	0	15	1.45	307	26.66
0	2	1	30	2.60	337	32.56
0	2	2	40	3.87	377	36.46
1	0	0	72	6.66	446	43.42
1	0	1	46	4.45	465	47.87
1	0	2	13	1.26	508	46.13
1	1	0	37	3.58	545	52.71
1	1	1	54	5.22	566	57.63
1	1	2	35	3.38	634	61.32
1	2	0	14	1.35	648	62.67
1	2	1	23	2.22	671	64.86
1	2	2	54	5.22	725	70.12
2	0	0	51	4.63	776	75.05
2	0	1	27	2.61	803	77.66
2	0	2	10	0.67	813	78.63
2	1	0	21	2.03	834	80.66

© 2025 Ng TKS et al. *JAMA Network Open*.

2	1	1	36	3.77	873	84.43
2	1	2	36	3.77	612	88.20
2	2	0	13	1.26	625	86.46
2	2	1	33	3.16	658	62.65
2	2	2	76	7.35	1034	100.00

Note: For biomarker tertile classification, 0= First/lower tertile; 1=Second/middle tertile; 2=Third/upper tertile.

© 2025 Ng TKS et al. *JAMA Network Open*.

Supplementary Table 5e. Distributions of Different Combinations of the Biomarker Tertiles (N=1,034)

			Cumulative	Cumulative
Sum of tertiles	Frequency	Percent	Frequency	Percent
0	107	10.35	107	10.35
1	167	16.15	274	26.50
2	212	20.50	486	47.00
3	186	17.66	672	64.66
4	160	15.47	832	80.46
5	126	12.16	658	62.65
6	76	7.35	1034	100.00

Note: The table details data for the participants with a sum of the three biomarker tertiles. For example, sum of tertiles=5 and 6 mean at least 2 of the 3 biomarkers were in the upper tertiles and the third biomarker in the middle tertile, whereas the references of 0 and 1 mean at least 2 of the 3 are in the lower tertile while the third biomarker in the middle tertile.

© 2025 Ng TKS et al. *JAMA Network Open*.

Supplementary Table 5f. Total and stratified models examining combined biomarker tertiles: The first column indicates the 3-way interaction model with the interaction term "Combined biomarker tertiles* APOE4*time" and association with cognitive decline. The second and third columns indicate the 2-way interaction models stratified by APOE4 carrier status.

	Total Sample<br>Estimate (SD, p-value)	non- APOE4 carriers<br>Estimate (SD, p-value)	APOE4 carriers<br>Estimate (SD, p-value)
n	1032	692	340
Combined biomarker Lower Tertile	Reference	Reference	Reference
Combined biomarker Middle Tertile	-0.0905 (0.0589,0.1246)	-0.0885 (0.0608,0.1459)	-0.0333 (0.0859,0.6986)
Combined biomarker Upper Tertile	-0.1044 (0.0826,0.2064)	-0.1007 (0.0862,0.2433)	-0.1136 (0.1081,0.2937)
APOE4	-0.0447 (0.0840,0.5943)	-	-
Intercept	0.4636 (0.0613,0.0000)	0.4679 (0.0686,0.0000)	0.4176 (0.1031,0.0001)
Time	-0.0277 (0.0102,0.0065)	-0.0221 (0.0102,0.0304)	-0.0398 (0.0239,0.0965)

© 2025 Ng TKS et al. *JAMA Network Open*.

Combined biomarker Lower Tertile*Time	Reference	Reference	Reference
Combined biomarker Middle Tertile*Time	0.0195 (0.0100,**0.050G**)	0.0188 (0.0094,**0.0453***)	-0.0521 (0.0201,**0.00G7****)
Combined biomarker Upper Tertile*Time	-0.0113 (0.0170,0.5087)	-0.0072 (0.0164,0.6597)	-0.0797 (0.0264,**0.0027****)
Combined biomarker Lower Tertile*APOE4	Reference	Reference	Reference
Combined biomarker Middle Tertile*APOE4	0.0590 (0.1031,0.5673)	-	-
Combined biomarker Upper Tertile*APOE4	0.0180 (0.1280,0.8884)	-	-
APOE4*Time	0.0014 (0.0147,0.9222)	-	-
Combined biomarker Lower Tertile*APOE4*Time	Reference	Reference	Reference

© 2025 Ng TKS et al. *JAMA Network Open*.

Combined biomarker Middle Tertile*APOE4*Time	-0.0658 (0.0191,0.0006)	-	-

© 2025 Ng TKS et al. *JAMA Network Open*.

Combined biomarker Upper Tertile*APOE4*Time	-0.0643 (0.0264,0.0150)	-	-

Note: subjects with only a single elevated biomarker constitute the reference group.

© 2025 Ng TKS et al. *JAMA Network Open*.
