# Supplementary material for: APOE4, Blood Neurodegenerative Biomarkers, and Cognitive Decline in Community-Dwelling Older Adults
Source: JAMA Netw Open. 2025 May 7;8(5):e258903. doi: 10.1001/jamanetworkopen.2025.8903 (PMC12059971; doi:10.1001/jamanetworkopen.2025.8903)
Supplement: Supplement 2. — Data Sharing Statement [file jamanetwopen-e258903-s002.pdf]

Data Sharing Statement

Ng. *APOE4,* Blood Neurodegenerative Biomarkers, and Cognitive Decline in Community-Dwelling Older Adults. *JAMA Network Open*. Published online May 7, 2025. doi:10.1001/jamanetworkopen.2025.8903

Data
Data available: Yes
Data types: Deidentified participant data
How to access data: The Institutional Review Board of the Rush University Medical Center approved the study protocols, and all participants provided written consent for blood and DNA collection, population interviews, and clinical evaluations. Data that support study findings are available through data request and a data use agreement from our research resource data portal, https://riha.rush.edu/dataportal.html.
When available: With publication

Supporting Documents
Document types: None

Additional Information
Who can access the data: researchers whose proposed use of the data has been approved
Types of analyses: researchers whose proposed use of the data has been approved)
Mechanisms of data availability: researchers whose proposed use of the data has been approved)
